# Serum Tumor Markers and Outcomes in Patients With Appendiceal Adenocarcinoma

**DOI:** 10.1001/jamanetworkopen.2024.0260

**Published:** 2024-02-28

**Authors:** Abdelrahman Yousef, Mahmoud Yousef, Mohammad A. Zeineddine, Aditya More, Mohammad Fanaeian, Saikat Chowdhury, Mark Knafl, Paul Edelkamp, Ichiaki Ito, Yue Gu, Vinay Pattalachinti, Zahra Alavi Naini, Fadl A. Zeineddine, Jennifer Peterson, Kristin Alfaro, Wai Chin Foo, Jeff Jin, Neal Bhutiani, Victoria Higbie, Christopher P. Scally, Bryan Kee, Scott Kopetz, Drew Goldstein, Madeleine Strach, Andrew Williamson, Omer Aziz, Jorge Barriuso, Abhineet Uppal, Michael G. White, Beth Helmink, Keith F. Fournier, Kanwal P. Raghav, Melissa W. Taggart, Michael J. Overman, John Paul Shen

**Affiliations:** 1 Department of Gastrointestinal Medical Oncology, University of Texas MD Anderson Cancer Center, Houston; 2 Department of Genomic Medicine, University of Texas MD Anderson Cancer Center, Houston; 3 Department of Data Engineering and Analytics, University of Texas MD Anderson Cancer Center, Houston; 4 Department of Pathology, University of Texas MD Anderson Cancer Center, Houston; 5 Department of Enterprise Development and Integration, University of Texas MD Anderson Cancer Center, Houston; 6 Department of Colon and Rectal Surgery, University of Texas MD Anderson Cancer Center, Houston; 7 Department of Surgical Oncology, University of Texas MD Anderson Cancer Center, Houston; 8 Department of Internal Medicine, Houston Methodist Hospital, Houston, Texas; 9 Palantir Technologies, Denver, Colorado; 10 Colorectal and Peritoneal Oncology Centre, The Christie NHS Foundation Trust, Manchester, United Kingdom; 11 Division of Cancer Sciences, Faculty of Biology, Medicine and Health, School of Medical Sciences, University of Manchester, Manchester, United Kingdom; 12 Faculty of Medicine and Health, The University of Sydney, Darlington, Victoria, Australia; 13 Department of Medical Oncology, The Christie National Health Service Foundation Trust, Manchester, United Kingdom

## Abstract

**Importance:**

Serum tumor markers carcinoembryonic antigen (CEA), carbohydrate antigen 19-9 (CA19-9), and cancer antigen 125 (CA125) have been useful in the management of gastrointestinal and gynecological cancers; however, there is limited information regarding their utility in patients with appendiceal adenocarcinoma.

**Objective:**

To assess the association of serum tumor markers (CEA, CA19-9, and CA125) with clinical outcomes and pathologic and molecular features in patients with appendiceal adenocarcinoma.

**Design, Setting, and Participants:**

This is a retrospective cohort study at a single tertiary care comprehensive cancer center. The median (IQR) follow-up time was 52 (21-101) months. Software was used to query the MD Anderson internal patient database to identify patients with a diagnosis of appendiceal adenocarcinoma and at least 1 tumor marker measured at MD Anderson between March 2016 and May 2023. Data were analyzed from January to December 2023.

**Main Outcomes and Measures:**

Association of serum tumor markers with survival in patients with appendiceal adenocarcinoma. Cox proportional hazards regression analyses were also performed to assess associations between clinical factors (serum tumor marker levels, demographics, and patient and disease characteristics) and patient outcomes (overall survival).

**Results:**

A total of 1338 patients with appendiceal adenocarcinoma were included, with a median (range) age at diagnosis of 56.5 (22.3-89.6) years. The majority of the patients had metastatic disease (1080 patients [80.7%]). CEA was elevated in 742 of the patients tested (56%), while CA19-9 and CA125 were elevated in 381 patients (34%) and 312 patients (27%), respectively. Individually, elevation of CEA, CA19-9, or CA125 were associated with worse 5-year survival; elevated vs normal was 81% vs 95% for CEA (hazard ratio [HR], 4.0; 95% CI, 2.9-5.6), 84% vs 92% for CA19-9 (HR, 2.2; 95% CI, 1.4-3.4), and 69% vs 93% for CA125 (HR, 4.6; 95% CI, 2.7-7.8) (*P* < .001 for all). Quantitative evaluation of tumor markers was associated with outcomes. Patients with highly elevated (top 10th percentile) CEA, CA19-9, or CA125 had markedly worse survival, with 5-year survival rates of 59% for CEA (HR, 9.8; 95% CI, 5.3-18.0), 64% for CA19-9 (HR, 6.0; 95% CI, 3.0-11.7), and 57% for CA125 (HR, 7.6; 95% CI, 3.5-16.5) (*P* < .001 for all). Although metastatic tumors had higher levels of all tumor markers, when restricting survival analysis to 1080 patients with metastatic disease, elevated CEA, CA19-9, or CA125 were all still associated worse survival (HR for CEA, 3.4; 95% CI, 2.5-4.8; *P* < .001; HR for CA19-9, 1.8; 95% CI, 1.2-2.7; *P* = .002; and HR for CA125, 3.9; 95% CI, 2.4-6.4; *P* < .001). Interestingly, tumor grade was not associated with CEA or CA19-9 level, while CA-125 was slightly higher in high-grade tumors relative to low-grade tumors (mean value, 18.3 vs 15.0; difference, 3.3; 95% CI, 0.9-3.7; *P* < .001). Multivariable analysis identified an incremental increase in the risk of death with an increase in the number of elevated tumor markers, with an 11-fold increased risk of death in patients with all 3 tumor markers elevated relative to those with none elevated. Somatic mutations in *KRAS* and *GNAS* were associated with significantly higher levels of CEA and CA19-9.

**Conclusions and Relevance:**

In this retrospective study of serum tumor markers in patients with appendiceal adenocarcinoma, CEA, CA19-9, and CA125 were associated with overall survival in appendiceal adenocarcinoma. Given their value, all 3 biomarkers should be included in the initial workup of patients with a diagnosis of appendiceal adenocarcinoma.

## Key Points

**Question:**

Are serum tumor markers carcinoembryonic antigen (CEA), carbohydrate antigen 19-9 (CA19-9), or cancer antigen 125 (CA125) associated with outcomes in patients with appendiceal adenocarcinoma?

**Findings:**

In this cohort study of 1338 patients, elevation of CEA, CA19-9, or CA125 was associated with significantly worse 5-year survival; 81% vs 95%, 84% vs 92%, and 69% vs 93% elevated vs normal, respectively. Moreover, quantitative evaluation of tumor markers was associated with outcomes.

**Meaning:**

These findings suggest elevated levels of CEA, CA19-9, and CA125 are associated with overall survival in appendiceal adenocarcinoma, highlighting the importance of including all 3 biomarkers in the initial workup of patients with this disease.

## Teaser

This cohort study evaluates the association of serum tumor markers with clinical outcomes and pathologic and molecular features in patients with appendiceal adenocarcinoma.

## Introduction

Appendiceal adenocarcinoma (AA) is a heterogenous disease, with marked contrast in the natural history of low-grade and high-grade tumors (5-year overall survival [OS] 68% for low-grade vs 7% for high-grade).^1,2,3,4^ Unfortunately, for the majority of patients, there is already metastatic disease at the time of diagnosis.^5,6^ Metastatic spread of AA is almost exclusively limited to the peritoneal cavity, causing the clinical syndrome pseudomyxoma peritonei (PMP), in which the peritoneal surfaces and omentum are involved with diffuse gelatinous, mucinous implants.^7,8^ However, PMP progression is difficult to measure with traditional cross-sectional imaging as it frequently exists as a contiguous, erratically shaped area in the peritoneal cavity. In addition, current Response Evaluation Criteria in Solid Tumor (RECIST) criteria do not consider mucinous or cystic disease as measurable. For these reasons, standard RECIST criteria are poorly applicable to AA.^9^ Moreover, AA is a slowly progressive disease, and classically defined thresholds for determining changes in disease extent (typically ≥20% increase) may take years to occur. For these reasons, having a more dependable method to distinguish patients with AA who are more or less likely to have favorable disease outcomes would be highly advantageous for guiding patient discussions and treatment options.

Serum tumor markers (TMs) are commonly used for aid in evaluating diagnosis, prognosis, and treatment response in different types of malignant neoplasms.^10,11^ Three TMs have been well established in gastrointestinal cancers: carcinoembryonic antigen (CEA), carbohydrate antigen 19-9 (CA19-9), and cancer antigen 125 (CA125).^12,13,14^ These TMs have been associated with metastatic dissemination of tumor cells.^15,16,17^ While serum markers have been useful in detecting gastrointestinal tumors, there is a lack of information regarding their efficacy in patients with AA. Researchers have suggested using CEA as a potential TM based on its utility in detection and management of colon cancer.^12^ However, studies conducted thus far have not established a definitive and consistent correlation between any of the TMs and AA outcomes.^18,19,20,21^

The objectives of this study were to investigate the association between serum TM levels and clinical outcomes, as well as pathologic and molecular features across the spectrum of AA. We hypothesized that elevation in any of the 3 TM levels would be associated with a decline in 5-year survival, independent of other factors.^10,11,22,23,24^

## Methods

Under approved institutional review board (IRB) protocol from MD Anderson Cancer Center (MDACC), the Palantir Foundry software system (Palantir)^25,26,27,28^ was used to query the MDACC internal patient database to identify patients with a diagnosis of AA and at least 1 TM measured at MDACC between 2016 to 2023 for inclusion in this retrospective study. The platform aids in the integration, analysis, extraction, and transformation of clinical data, allowing the many elements of the electronic health record to be merged into a dataset amenable to research analyses. The data cutoff point was May 12, 2023. The IRB waived consent under 45 CFR 46.116(F) as the research involves no more than minimal risk of harm to the participants. This study followed the Strengthening the Reporting of Observational Studies in Epidemiology (STROBE) reporting guideline for cohort studies.

### Statistical Analysis

The value of each TM measured was categorized as normal, elevated, or highly elevated. TM levels below the laboratory upper limit of normal (CEA ≥3 ng/mL, CA19-9 ≥35 U/mL, and CA125 ≥35 U/mL from March 2016 to March 2018, and CEA >3.8 ng/mL, CA19-9 >35 U/mL, and CA125 >38 U/mL from April 2018 to May 2023, as the TM panels changed in 2018) were defined as normal. TM levels above the laboratory upper limit of normal were defined as elevated; while levels in the top tenth percentile for each respective TM (>99.8 ng/mL for CEA, >338.6 U/mL for CA19-9, and >99.0 U/mL for CA125) were defined as highly elevated. For patients with more than 1 measurement for each TM, the highest measurement was considered for analysis. Other clinical information collected through the platform included patient demographics, histopathology, tumor grade, surgical history, and tumor somatic mutation profiles for patients who had next-generation sequencing (NGS) performed at MDACC. Patients’ race and ethnicity were self-reported and were assessed to better understand if race is associated with survival or disease outcomes. Histologic classification and grade were collected from patients’ pathology records. Pathologic diagnosis was determined by a team of expert pathologists (including M.W.T. and W.C.F.) using a 3-tier classification.^29^ Well-differentiated and well- to moderately differentiated tumors were considered low-grade tumors, while moderately differentiated, moderately to poorly differentiated, and poorly differentiated tumors were considered high-grade tumors. Low-grade tumors lacked high cellularity, invasive implants, or significant cytologic atypia. High-grade tumors exhibited invasive implants, cytologic atypia, and signet ring cells. OS was defined as the time from initial diagnosis with appendiceal cancer until death. From the main cohort, a subcohort of patients who received chemotherapy at MDACC, had CEA measured 30 days before chemotherapy start date, and had CEA measured again within 180 days after chemotherapy were identified to evaluate the utility of TM measurement in assessing response to treatment; OS was assessed from chemotherapy start date until death. Significance was set at a *P *value of .05. All statistical analysis was performed using GraphPad Prism software version 9.0.0 for Windows (GraphPad Software) and RStudio (RStudio Team). Data were analyzed from January to December 2023. For additional details regarding methods, see eAppendix in Supplement 1.

## Results

### Patient Characteristics

Between 2016 to 2023, a total of 1338 patients who had a diagnosis of AA and at least 1 TM (CEA, CA19-9, or CA125) measured at MD Anderson were identified (eFigure 1 in Supplement 1). The median (IQR) follow-up time from AA diagnosis was 52 (21-101) months and median (range) age of the patients at diagnosis was 56.5 (22.3-89.6) years (Table). The median OS was not yet reached, while 5-year and 10-year OS were 85% and 75%, respectively. The study population included slightly more female patients (753 patients [56.3%]), and 37 self-reported reported Asian race (2.8%), 81 self-reported Black race (6.1%), and 1067 patients self-reported White race (79.7%). Most of the patients in our study had stage IV metastatic disease (1080 patients [80.7%]). A total of 693 patients (52.4%) had mucinous tumors; 130 had colonic (9.8%), 93 had goblet cell (7.0%), 221 had signet ring cell (16.7%), and 147 had a mix of signet ring and goblet cell (11.1%) histopathology. Overall, 766 patients (57.2%) had high-grade tumors (moderately or poorly differentiated) (Table; eFigure 2 in Supplement 1).

**Table.** Patient Characteristics of MD Anderson Cohort

Patient characteristics	Patients, No. (%) (N = 1338)
Age at diagnosis, median (range), y	56.5 (22.3-89.6)
Race and ethnicity	
Asian	37 (2.8)
Black or African	81 (6.1)
Hispanic	127 (9.5)
White	1067 (79.7)
Other^a^	26 (1.9)
Sex	
Female	753 (56.3)
Male	585 (43.7)
Smoking status	
Never	897 (67.0)
Smoker	53 (4.0)
Former	299 (22.3)
Missing	89 (6.7)
Alcohol use status	
Yes	743 (55.5)
No	478 (35.7)
Missing	117 (8.7)
Histologic grade	
Well differentiated	465 (34.8)
Moderately differentiated	410 (30.6)
Poorly differentiated	412 (30.8)
Missing	51 (3.8)
Histologic grade binary	
Low grade	521 (38.9)
High grade	766 (57.2)
Missing	51 (3.8)
Histopathology	
Mucinous	693 (52.4)
Colonic	130 (9.8)
Goblet cell	93 (7.0)
Signet ring cell	221 (16.7)
Mix of goblet and signet	147 (11.1)
Missing	54 (3.0)
Disease metastatic state	
Localized disease (stage I, II, III)	258 (19.3)
Metastatic disease (stage IV)	1080 (80.7)
No. of elevated tumor markers	
0	485 (36.2)
1	419 (31.3)
2	286 (21.4)
3	148 (11.1)
CEA	
Normal	589 (44.0)
Elevated	609 (45.5)
Highly elevated	133 (9.9)
Not tested	7 (0.5)
CA19-9	
Normal	751 (56.1)
Elevated	268 (20.0)
Highly elevated	113 (8.4)
Not tested	206 (15.4)
CA125	
Normal	853 (63.8)
Elevated	196 (14.6)
Highly elevated	116 (8.7)
Not tested	173 (12.9)
Overall survival	
Median (range), mo	Not yet reached (0-272)

Abbreviations: CA125, cancer antigen 125; CA19-9, carbohydrate antigen 19-9; CEA, carcinoembryonic antigen.

^a^ Other race includes Native Hawaiian, Other Pacific Islander, American Indian, or Alaska Native.

In the validation cohort (The Christie NHS Foundation Trust Cohort), 216 patients with AA were identified. A total of 141 had mucinous adenocarcinoma, 71 had adenocarcinoma not otherwise specified, and 4 had signet ring cell carcinoma.^30^ Baseline demographics and clinical characteristics are summarized in eTable 1 in Supplement 1. At initial diagnosis, 69 patients (32%) had localized disease on CT imaging and preoperative pathology, and 147 (68%) had metastatic disease. At a median (range) follow-up of 56 (1-286) months, the median OS was 122 (95% CI, 62-182) months with 5-year and 10-year OS of 63% and 51%, respectively.

### Tumor Marker Assessment

CEA was the most frequently tested TM (1331 patients); CA19-9 and CA125 were also evaluated in the majority of patients (1132 and 1165 patients, respectively) (eTable 2 in Supplement 1). CEA was elevated 742 of the patients tested (56%, including highly elevated levels), while CA19-9 was elevated in 381 patients (34%, including highly elevated levels) and CA125 in 312 of the patients tested (27%, including highly elevated levels); 148 patients (11%) had elevated levels of all 3 TMs (eTable 2, eTable 3, and eFigure 3 in Supplement 1). There has been a trend of increased testing of all 3 markers at time of diagnosis starting in 2018 (eFigure 4 in Supplement 1).

**Figure 1.**
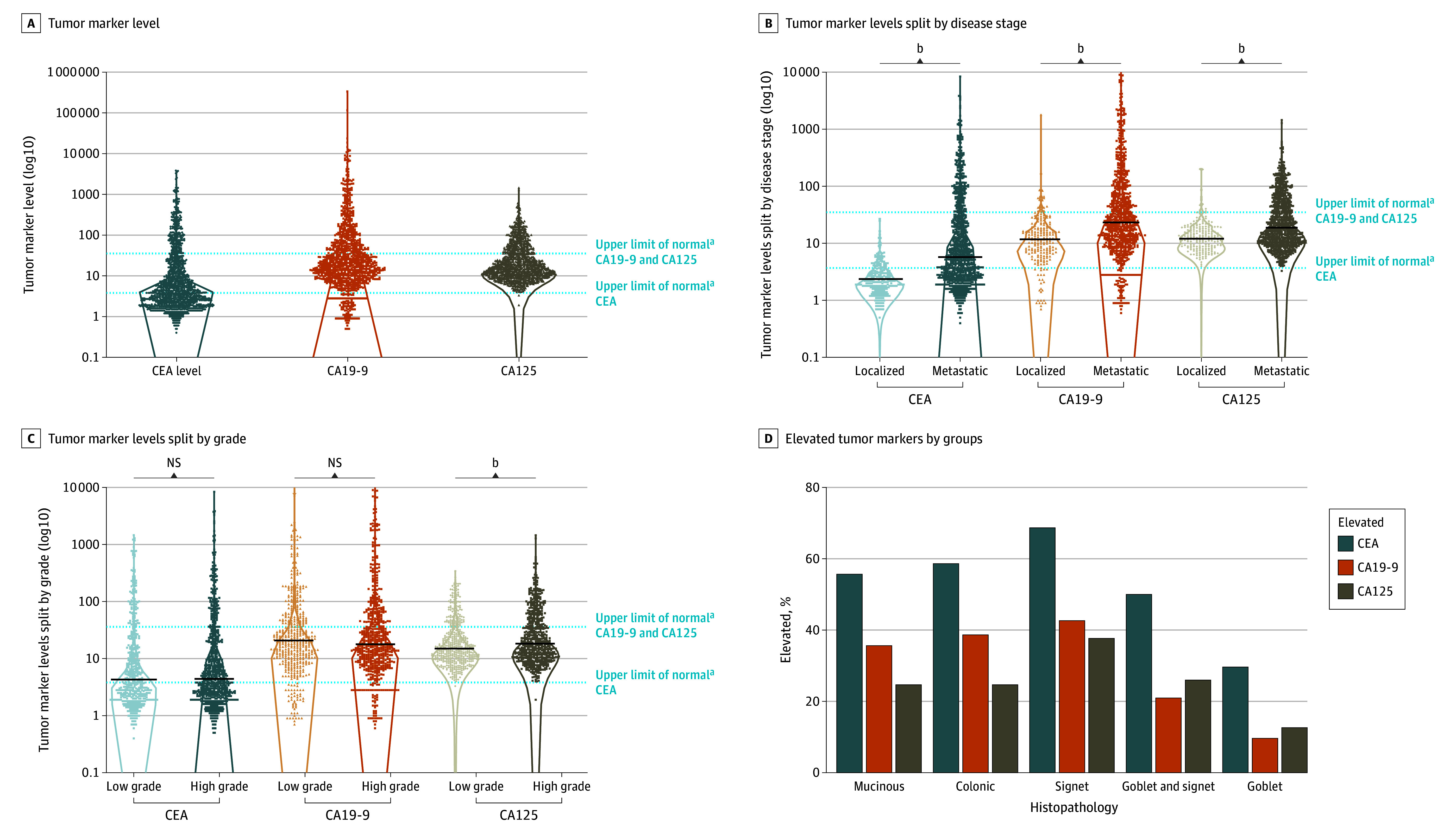
Distribution of Carcinoembryonic Antigen (CEA), Carbohydrate Antigen 19-9 (CA19-9), and Cancer Antigen (CA125) Tumor Marker Levels A, Distribution of all patients CEA, CA19-9, and CA125 tumor markers levels; each point represents one patient. B, Distribution of all patients CEA, CA19-9, and CA125 tumor markers levels split by disease stage (metastatic vs localized); lines represent median levels. C, Distribution of all patients CEA, CA19-9, and CA125 tumor markers levels split by grade; lines represent median levels. D, Percentage of elevated tumor markers in different histopathological groups. ^a^Laboratory upper limit of normal (CEA 3.8 ng/mL, CA19-9 35 U/mL, and CA125 38 U/mL). ^b^*P* < .001.

CA19-9 and CA125 levels were statistically equivalent between male and female patients (eFigure 5 in Supplement 1). CEA, CA19-9, and CA125 were significantly higher in patients with metastatic disease compared with patients with localized disease (median [IQR] of metastatic vs localized for CEA, 5.8 [2.6-26.9] vs 2.2 [1.6-3.4]; difference, 3.6; 95% CI, 2.5 to 4.3 ng/mL; for CA19-9, 23.3 [10.1-77.3] vs 11.6 [6.6-19.3]; difference, 11.7; 95% CI, 8.0 to 14.7 U/mL; and for CA125, 18.5 [10.5-50.0] vs 12.5 [8.8-18.5]; difference, 6.0; 95% CI, 4.1 to 8.3 U/mL) (*P* < .001 for all) (Figure 1B). CEA and CA19-9 were statistically equivalent for high- and low-grade tumors (CEA difference, 0.1; 95% CI, −0.2 to 0.6; *P* = .40; CA19-9 difference, −3.0; 95% CI, −3.0 to 1.5; *P* = .55), CA125 was slightly higher in high-grade tumors (median [IQR] of high grade vs low grade, 18.3 [10.5-48.8] vs 15.0 [9.8-31.3]; difference, 3.25; 95% CI, 0.9 to 3.7; *P* < .001) (Figure 1C; eTable 4 in Supplement 1). Comparing across histologic findings, all 3 TMs were most often elevated in patients with signet ring cell histologic findings (CEA, 153 patients [69%]; CA19-9, 77 patients [43%]; and CA125, 73 patients [38%]; Kruskal-Wallis statistic, 47.4 for CEA, 34.0 for CA19-9, and 29.9 for CA125; *P* < .001 for each) and least likely to be elevated in Goblet cell adenocarcinoma (Figure 1D; eFigure 6, eTable 5, eTable 6, and eTable 7 in Supplement 1). When measured on the same day, CEA and CA19-9 levels were highly correlated (*r* = 0.63; *P* < .001), but there was a subset of patients with highly elevated CEA but normal CA19-9, consistent with the fact that the carbohydrate CA19-9 cannot be produced in patients who genetically lack the Lewis antigen A.^31^ Both CEA and CA19-9 were correlated with CA125 but to a lesser degree (CEA *r* = 0.29; CA19-9 *r* = 0.25; *P* < .001 for each) (eFigure 7 in Supplement 1). In patients who had all 3 markers measured (1112 patients), isolated CEA elevation was more common (207 patients [18.6%]) than CA19-9 (40 patients [3.6%]) or CA125 (43 patients [3.9%]); all 3 TMs were elevated in 148 patients (13.3%) (eFigure 3 in Supplement 1).^32^

### Outcomes

**Figure 2.**
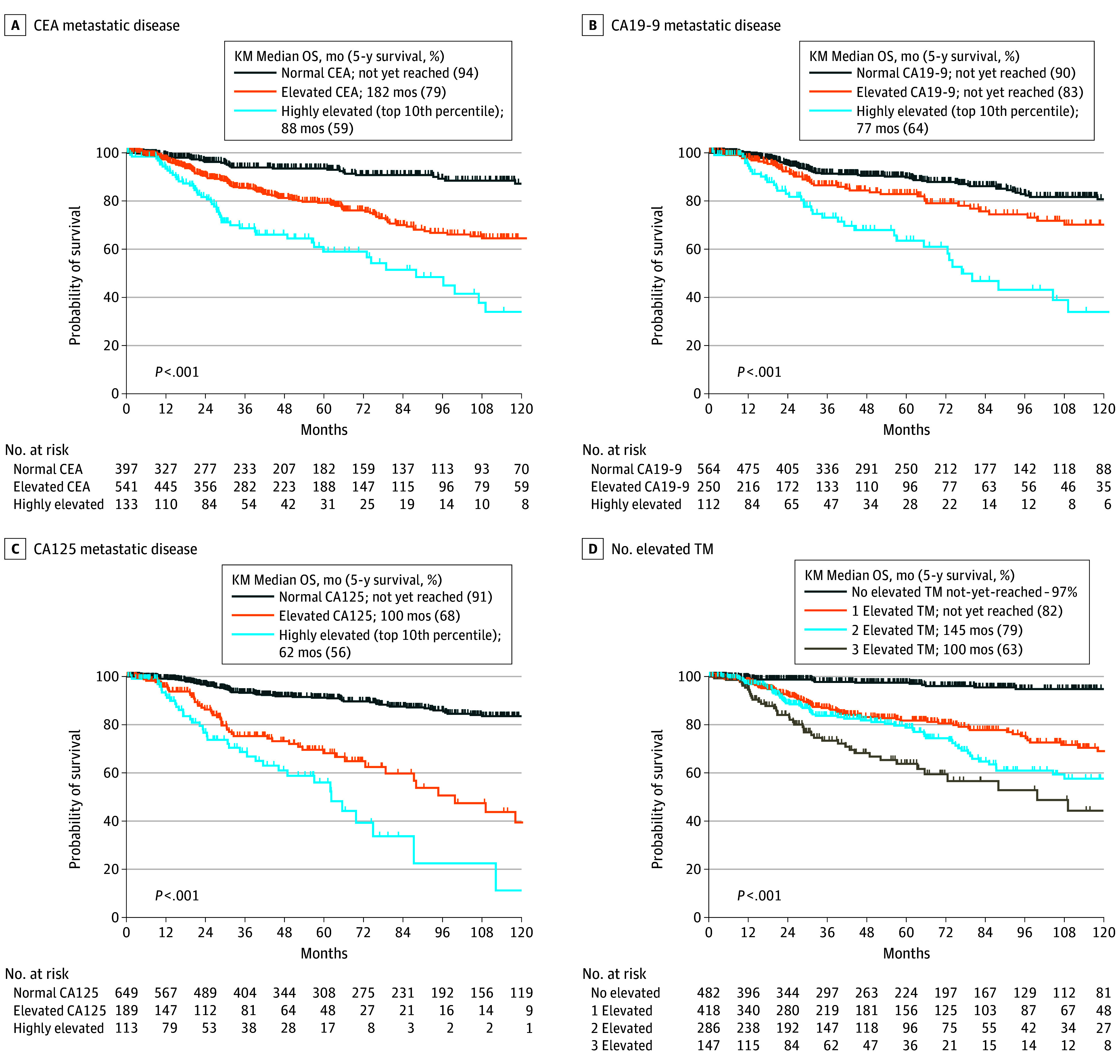
Survival Plots of Patients With Metastatic Disease by Tumor Markers A, Stage IV metastatic disease patients with normal, elevated, and highly elevated levels of carcinoembryonic antigen (CEA). B, Stage IV metastatic disease patients with normal, elevated, and highly elevated levels of carbohydrate antigen 19-9 (CA19-9). C, Stage IV metastatic disease patients with normal, elevated, and highly elevated levels of cancer antigen 125 (CA125). D, All patients with number of elevated tumor markers. KM indicates Kaplan-Meier; OS, overall survival.

**Figure 3.**
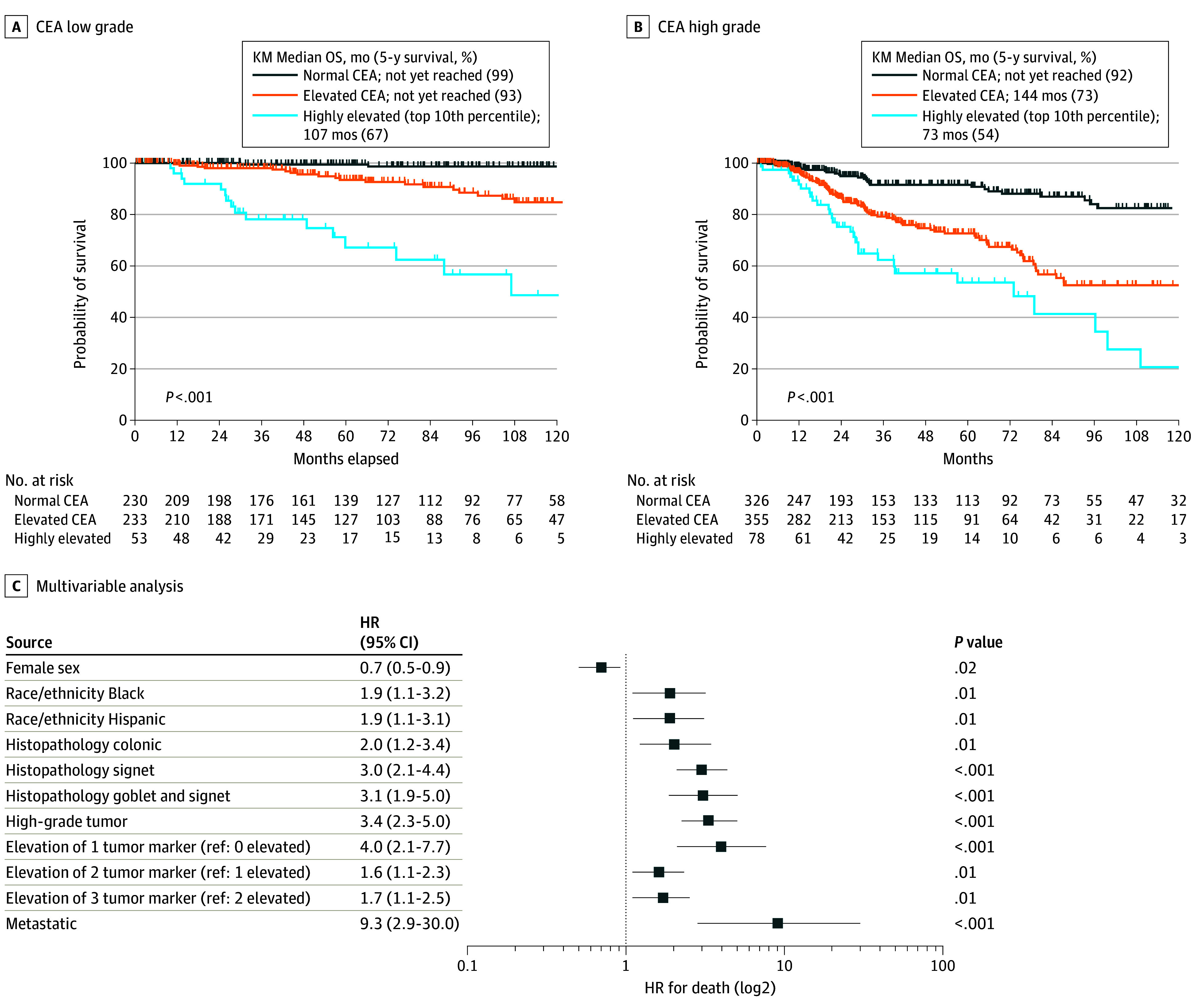
Survival Plots for Patients With Low and High Tumor Grades A, Patients with low grade tumor for normal, elevated, and highly elevated levels of carcinoembryonic antigen (CEA). B, Patients with high grade tumor for normal, elevated, and highly elevated levels of CEA. C, Multivariable analysis showing HR for death in all patients on a log2 axis. KM indicates Kaplan-Meier; OS, overall survival.

Kaplan-Meier survival analysis of OS by TM marker level (normal, elevated, or highly elevated) demonstrated that CEA, CA19-9, and CA125 were all associated with OS (eFigure 8 in Supplement 1). Compared with 5-year OS of 92% to 95% in patients with normal values for each TM, 5-year OS with elevated CEA was 81% (hazard ratio, [HR], 4.0; 95% CI, 2.9-5.6), CA19-9 was 84% (HR, 2.2; 95% CI, 1.4-3.4), and CA125 was 69% (HR, 4.6; 95% CI, 2.7-7.8) (*P* < .001 for all). Moreover, 5-year OS for those with highly elevated CEA was 59% (HR, 9.8; 95% CI, 5.3-18.0), CA19-9 was 64% (HR, 6.0; 95% CI, 3.0-11.7), and CA125 was 57% (HR, 7.6; 95% CI, 3.5-16.5) (*P* < .001 for all). Given the association of metastasis with increased TM levels, the survival analysis for each TM was repeated, restricting to only patients with metastatic disease (1080 patients). Elevated levels of all TMs remained associated with OS (HR elevated vs normal for CEA, 3.4; 95% CI, 2.5-4.8; *P* < .001; HR for CA19-9, 1.8; 95% CI, 1.2-2.7; *P* = .002; HR for CA125, 3.9; 95% CI, 2.4-6.4; *P* < .001) (Figure 2A, Figure 2B, and Figure 2C) as well as highly elevated levels (HR highly elevated vs normal for CEA, 7.4; 95% CI, 4.2-12.8; HR for CA19-9, 4.7; 95% CI, 2.6-8.5; HR for CA125, 6.4; 95% CI, 3.1-13.1) (*P* < .001 for all). Survival analysis for each TM was again repeated controlling for tumor grade. Elevated levels of all TMs remained associated with OS in both low-grade and high-grade tumor subgroups (Figure 3A and Figure 3B; eFigure 9 in Supplement 1). As a further control, analysis was restricted to those patients with TMs measured within the initial 6 months from the date of diagnosis (560 for CEA, 461 for CA19-9, and 475 for CA125) to allow for assessment of TMs at time of diagnosis. Again, survival analysis by TM level (normal, elevated, or highly elevated) demonstrated that CEA, CA19-9, and CA125 were all associated with OS (HR, 2.4; 95% CI, 1.4-3.9 for elevated CEA; HR, 3.6; 95% CI, 1.4-9.3 for highly elevated CEA; HR, 1.8; 95% CI, 0.9-3.7 for elevated CA19-9; HR, 4.5; 95% CI, 2.1-9.8 for highly elevated CA19-9; HR, 2.4; 95% CI, 1.2-4.6 for elevated CA125; and HR, 4.0; 95% CI, 2.0-7.8 for highly elevated CA125) (*P* < .001 for all) (eFigure 10 in Supplement 1).

In the validation cohort, similar to MD Anderson cohort, Kaplan-Meier survival analysis of OS by TM level (normal, elevated, or highly elevated) demonstrated that CEA, CA19-9, and CA125 were all associated with OS; however, only the HR for highly elevated CA19-9 achieved statistical significance (eFigure 11 in Supplement 1). Compared with 5-year OS of 75% to 84% in the patients with normal values for each TM, 5-year OS with elevated CEA was 43% (HR, 3.5; 95% CI, 2.0-6.3; *P* < .001), CA19-9 was 59% (HR, 1.6; 95% CI, 0.9-3.0; *P* = .09), and CA125 was 57% (HR, 2.1; 95% CI, 1.1-4.2; *P* = .006). Moreover, 5-year OS for those with highly elevated CEA was 28% (HR, 5.8; 95% CI, 2.1-15.8), CA19-9 was 33% (HR, 4.9; 95% CI, 1.7-14.0), and CA125 was 29% (HR, 4.1; 95% CI, 1.6-10.0) (*P* < .001 for all). (eFigure 11 in Supplement 1). Given the association of metastasis with increased TM levels, the survival analysis for each TM was repeated restricting to the 147 patients with metastatic disease. Elevated levels of CEA remained associated with OS (HR, 2.4; 95% CI, 1.4-4.1; *P* = .001), while OS was not associated with CA19-9 and CA125 (HR for CA19-9, 1.3; 95% CI, 0.6-2.8; *P* = .49; HR for CA125, 1.5; 95% CI, 0.8-2.8; *P* = .18). However, highly elevated levels for all remained associated with OS (HR highly elevated vs normal, CEA, 3.3; 95% CI, 1.4-7.8; *P* < .001; HR for CA19-9, 3.0; 95% CI, 1.3-7.0; *P* < .001; HR for CA125, 2.6; 95% CI, 1.2-5.6; *P* = .001) (eFigure 11 in Supplement 1).

In multivariable analysis, after controlling for race and ethnicity, goblet or signet histologic findings, tumor grade, and tumor stage, elevated CEA (HR, 2.8; 95% CI, 1.7-4.9; *P* < .001), elevated CA19-9 (HR, 1.5; 95% CI 1.0-2.2; *P* = .03), and elevated CA125 (HR, 3.2; 95% CI 2.2-4.7; *P* < .001) remained significantly associated with decreased OS (eTable 8 in Supplement 1). Notably, when the same variables were modeled together with the number of elevated TMs, the incremental increase in the number of elevated TMs continued to worsen survival when the number of elevated TMs were compared with one another (HR, 1.7; 95% CI, 1.1-2.5; *P* = .01, for 3 vs 2 TMs elevated) (Figure 3C; eTable 9 in Supplement 1). Similar findings were observed in the subset of 560 patients who had their TMs measured within the first 6 months from diagnosis (eFigure 10 in Supplement 1).

In the subset of 121 patients who received chemotherapy at MDACC, 32 had a 2 ng/mL or more increase in CEA levels, and 89 had stable or decreased levels of CEA. There was a significant difference in median OS between the 2 groups; median OS for patients who had 2 ng/mL or less elevation in CEA was 10 vs 49 months for patients who had CEA levels that decreased or remained stable (HR, 3.0; 95% CI, 1.6-5.6; *P* < .001) (eFigure 12 in Supplement 1). These data suggest a possible role for serial TM measurement while receiving chemotherapy to assess for therapeutic response, important in appendiceal cancer given the difficulty in quantitating response by traditional imaging methods.^33^

**Figure 4.**
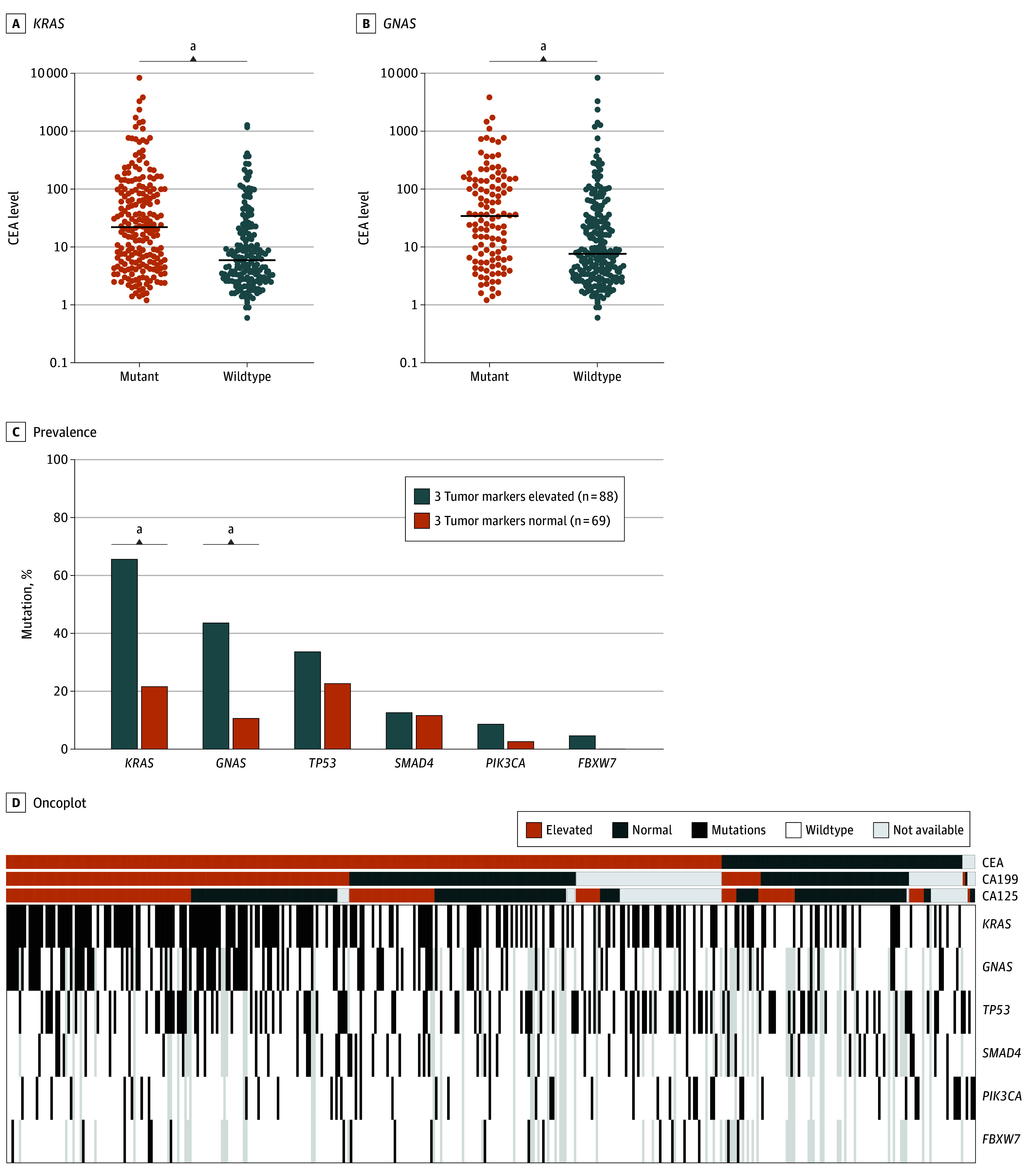
Somatic Mutation Landscape and Association With Tumor Markers A, *KRAS* somatic mutation vs wildtype with carcinoembryonic antigen (CEA) levels; lines represent the median levels. B, *GNAS* somatic mutation vs wildtype with CEA levels; lines represent the median levels. C, Prevalence of *KRAS, GNAS, TP53, SMAD4, PIK3CA,* and *FBXW7* (most common genes with somatic mutations in our cohort) somatic mutations in patients with the 3 tumor markers elevated (pan-elevated) vs patients with normal levels of the 3 tumor markers (pan-normal). D, 3 Tumor markers elevated levels on the left and normal levels on the right, and the somatic mutation status of *KRAS, GNAS, TP53, SMAD4, PIK3CA*, and *FBXW7.* ^a^*P* < .001.

A subset of 398 tumors were also profiled with a targeted somatic mutation panel; CEA and CA19-9 were more frequently elevated in patients with *KRAS* somatic mutation (79% vs 64% for CEA; odds ratio [OR], 2.0; 95% CI, 1.3-3.4; *P* = .003; 64% vs 33% for CA19-9; OR, 3.6; 95% CI, 2.2-5.7; *P* < .001) (eFigure 13 in Supplement 1). Similarly, both CEA and CA19-9 were more frequently elevated in patients with tumors with *GNAS* somatic mutations (88% vs 72% for CEA; OR, 2.8; 95% CI, 1.5-5.1; *P* = .001; 68% vs 43% for CA19-9; OR, 2.8; 95% CI, 1.7-4.7; *P* < .001) (eFigure 13 in Supplement 1). CEA and CA19-9 levels were higher in patients with tumors with *KRAS* somatic mutation (median [IQR], 22.0 [5.4-99] vs 6.0 [2.8-19.5]; *P* < .001 for CEA; and 80.4 [16.9-480.0] vs 18.9 [10.0-55.2]; *P* < .001 for CA19-9) (Figure 4A; eFigure 13 in Supplement 1) and tumors with *GNAS* somatic mutations (median [IQR], 34.3 [6.4-143.6] vs 7.6 [3.4-31.4]; *P* < .001 for CEA; and 94.0 [23.5-326.7] vs 26.2 [11.2-118.8]; *P* < .001 for CA19-9) (Figure 4B; eFigure 13 in Supplement 1). *TP53* somatic mutation was not associated with differences in TM level (eFigure 13 in Supplement 1). CA-125 levels were statistically equivalent regardless of somatic mutation status for *KRAS*, *GNAS*, and *TP53*. A total of 88 patients had elevated values for all 3 TMs (pan-elevated TM group), and 69 patients had normal values for all 3 TMs (the pan-normal TM group). Prevalence of *KRAS* and *GNAS* somatic mutations was significantly higher in the pan-elevated group than in the pan-normal group (66% vs 22% for *KRAS*; OR, 7.0; 95% CI, 3.3-14.4; and 44% vs 11% for *GNAS*; OR, 6.3; 95% CI, 2.6-15.9) (*P* < .001 for both) (Figure 4C). Finally, Figure 4D displays significant concurrent elevation between CEA and CA19-9 (OR, 9.4; 95% CI, 6.8-12.9; *P* < .001), CEA and CA125 (OR, 4.9; 95% CI, 3.6-6.7; *P* < .001), and CA19-9 and CA125 (OR, 4.1; 95% CI, 3.1-5.4; *P* < .001). In our cohort, *KRAS* and *GNAS* somatic mutations were the most common (52% and 33%, respectively).

## Discussion

This study represents the first comprehensive evaluation we know of the clinical utility of the tumor markers CEA, CA19-9, and CA125 in more than a thousand patients with appendiceal adenocarcinoma, establishing that each of the 3 are biomarkers associated with outcomes. In 2018 the Chicago Consensus Working Group for the first time developed guidelines for the treatment of appendiceal cancer; these endorsed the measurement of CEA, CA19-9, and CA125 in all patients with metastatic appendiceal cancer.^34^ However, in practice, even at this National Comprehensive Cancer Network–designated tertiary referral center, testing of all 3 tumor markers was not universal (eFigure 4 in Supplement 1). Here we show that using the combination of CEA, CA19-9, and CA125 can stratify patients with appendiceal adenocarcinoma into groups with 5-year survival ranging from 97% for those with no tumor markers elevated to 63% for those with all 3 markers elevated (Figure 2D). Our results are consistent with and expand upon multiple prior retrospective analyses in small cohorts^35,36^ and prior studies restricted to patients undergoing cytoreductive surgery^19,37^ which have suggested prognostic value of these tumor markers. Most prior studies of TMs in appendiceal cancer dichotomized each TM into elevated and normal categories; however, we find that treating each as a continuous variable retains important information. The distribution of values for each of CEA, CA19-9, and CA-125 were positively skewed but unimodal, so an arbitrary cutoff of the highest 10% was chosen to evaluate the survival association of highly elevated TM. We found that highly elevated CEA, CA19-9, and CA-125 had a greater association with poor survival, similar in magnitude to stage.

To assess the clinical utility of TMs in both operable and inoperable patients, we first evaluated their association with tumor stage, grade,^38,39^ histopathology, and somatic mutation profile. Although metastatic tumors had higher levels of all 3 TMs, the association of elevated TM with survival was independent of stage, sex, race, tumor grade, tumor histopathology. The analyses by tumor grade also highlighted the unique observation that patients with low-grade AA and normal CEA levels had a particularly good prognosis (99% survival at 5 years and 94% survival at 5 years in metastatic disease). This observation could be explained by early diagnosis in these patients when the tumor volume is insufficient to cause elevation of the TMs, which would be associated with an especially favorable outcome. This should be studied prospectively, but there could be a role for treatment deescalation in these patients, particularly considering recent prospective data showing 5-fluorouracil–based chemotherapy is ineffective in this patient population.^33^ Conversely, for patients with highly elevated tumor markers, the poor prognoses seen with current treatments, which have historically been chemotherapy designed for CRC, suggests that these patients be prioritized for clinical studies testing appendiceal cancer–specific therapies. The association with *KRAS* and *GNAS* somatic mutations and elevated levels of both CEA and CA19-9 suggests that high TM expression tumors may have intrinsically distinct biology compared with nonexpressing tumors, which may be indicative of different therapeutic vulnerabilities and should be explored in future studies.

Despite the notable distinctions between AA and CRC,^2,38^ the current American Joint Committee on Cancer (AJCC) staging system treats them similarly^40^ and does not provide meaningful stratification of survival outcome for AA. Specifically, under the current AJCC stage I to III, all have excellent survival, and there is wide variation in survival among patients classified as stage IV. The greater survival association seen with CEA, CA19-9, and CA125 suggests that these tumor markers should be included in appendiceal adenocarcinoma staging to stratify patients with metastatic disease in a similar manner to human chorionic gonadotropin, α fetoprotein, and lactate dyhydrogenase in germ cell tumors.^41^

### Limitations

The study is subject to several limitations. First, the retrospective design introduces inherent limitations in data collection and potential biases. Additionally, the low number of patients undergoing NGS analysis (only 30% of the cohort) limits the comprehensive assessment of genetic somatic mutations. Additionally, the selective approach in ordering tumor markers by different physicians may have led to the exclusion of certain patients from the cohort, potentially overrepresenting those with a more advanced disease stage. Another limitation is the lack of consideration for patients’ chemotherapy and cytoreductive surgery history. This omission is primarily due to the fact that many patients receive initial treatment at local hospitals before seeking care at our institution. Furthermore, the analysis did not investigate the differences in tumor marker levels among patients with localized disease (stage I, II, and III) due to incomplete stage data in our cohort. Despite these limitations, our study benefited from the ability to assemble a substantial cohort of 1338 patients with AA, accompanied by comprehensive clinical data and outcomes. To our knowledge, this represents the largest and most comprehensive patient cohort with AA ever assembled at a single institution, providing valuable insights into the disease and its management and enabled us to control for potential variations in clinical practice, which is particularly relevant in rare diseases such as AA.

## Conclusions

In conclusion, these data demonstrate the practical value of CEA, CA19-9, and CA125 in management of AA. These biomarkers are associated with overall survival for patients with AA. We suggest incorporating the measurement of these 3 TMs as a standard part of AA’s clinical management. Additionally, it is important to explore the potential role of TMs in AA’s tumor cell adhesion and disease progression to enhance our comprehension of the disease’s biological behavior.

## Notes

Supplement 1. **eAppendix.** Supplemental Methods **eTable 1.** Validation Cohort Patient Characteristics **eTable 2. **Tumor Marker Levels and Cutoffs for the Main Cohort **eTable 3. **Number of Tests Performed for Each TM per Patient **eTable 4. **Tumor Marker Levels and Cutoffs Split by Tumor Grade **eTable 5.** CEA Level and Cutoff Split by Histopathology **eTable 6. **CA19-9 Level and Cutoff Split by Histopathology **eTable 7. **CA125 Level and Cutoff Split by Histopathology **eTable 8. **Cox Proportional Hazards Regression Model A for Overall Survival **eTable 9. **Cox Proportional Hazards Regression Model B for Overall Survival **eFigure 1. **Study Flow Chart **eFigure 2. **Distribution of Patients **eFigure 3. **Tumor Markers **eFigure 4. **Percentage of Patients Tested by Year of Diagnosis For Each Tumor Marker **eFigure 5. **CEA, CA19-9, and CA125 by Sex **eFigure 6. **CEA, CA19-9, and CA125 by Histopathology **eFigure 7. **Correlation of CEA, CA19-9, and CA125 **eFigure 8. **Survival Probability for all Patients Stratified by Tumor Markers **eFigure 9. **Survival Probability by Grade **eFigure 10. **Survival Probability for CEA, CA19-9, and CA125 for Patients Tested Within the First 6 Months From Diagnosis **eFigure 11. **Survival Probability for CEA, CA19-9, and CA125 for All Patients vs Metastatic in the Validation Cohort (The Christie NHS Foundation Trust Cohort) **eFigure 12. **Survival Probability After Receiving Chemotherapy Stratified by CEA **eFigure 13. **Association of GNAS and KRAS Somatic Mutations With Tumor Markers

Supplement 2.
Data Sharing Statement
