# Supplementary material for: Serum Tumor Markers and Outcomes in Patients With Appendiceal Adenocarcinoma
Source: JAMA Netw Open. 2024 Feb 28;7(2):e240260. doi: 10.1001/jamanetworkopen.2024.0260 (PMC10902735; doi:10.1001/jamanetworkopen.2024.0260)
Supplement: Supplement 1. — eAppendix. Supplemental Methods eTable 1. Validation Cohort Patient Characteristics eTable 2. Tumor Marker Levels and Cutoffs for the Main Cohort eTable 3. Number of Tests Performed for Each TM per Patient eTable 4. Tumor Marker Levels and Cutoffs Split by Tumor Grade eTable 5. CEA Level and Cutoff Split by Histopathology eTable 6. CA19-9 Level and Cutoff Split by Histopathology eTable 7. CA125 Level and Cutoff Split by Histopathology eTable 8. Cox Proportional Hazards Regression Model A for Overall Survival eTable 9. Cox Proportional Hazards Regression Model B for Overall Survival eFigure 1. Study Flow Chart eFigure 2. Distribution of Patients eFigure 3. Tumor Markers eFigure 4. Percentage of Patients Tested by Year of Diagnosis For Each Tumor Marker eFigure 5. CEA, CA19-9, and CA125 by Sex eFigure 6. CEA, CA19-9, and CA125 by Histopathology eFigure 7. Correlation of CEA, CA19-9, and CA125 eFigure 8. Survival Probability for all Patients Stratified by Tumor Markers eFigure 9. Survival Probability by Grade eFigure 10. Survival Probability for CEA, CA19-9, and CA125 for Patients Tested Within the First 6 Months From Diagnosis eFigure 11. Survival Probability for CEA, CA19-9, and CA125 for All Patients vs Metastatic in the Validation Cohort (The Christie NHS Foundation Trust Cohort) eFigure 12. Survival Probability After Receiving Chemotherapy Stratified by CEA eFigure 13. Association of GNAS and KRAS Somatic Mutations With Tumor Markers [file jamanetwopen-e240260-s001.pdf]

Supplemental Online Content

Yousef A, Yousef M, Zeineddine MA, et al. Serum tumor markers and outcomes in patients with appendiceal adenocarcinoma. *JAMA Netw Open*. 2024;7(2):e240260. doi:10.1001/jamanetworkopen.2024.0260

This supplemental material has been provided by the authors to give readers additional information about their work

eAppendix. Supplemental Methods

eTable 1. Validation Cohort Patient Characteristics

eTable 2. Tumor Marker Levels and Cutoffs for the Main Cohort

eTable 3. Number of Tests Performed for Each TM per Patient

eTable 4. Tumor Marker Levels and Cutoffs Split by Tumor Grade

eTable 5. CEA Level and Cutoff Split by Histopathology

eTable 6. CA19-9 Level and Cutoff Split by Histopathology

eTable 7. CA125 Level and Cutoff Split by Histopathology

eTable 8. Cox Proportional Hazards Regression Model A for Overall Survival

eTable 9. Cox Proportional Hazards Regression Model B for Overall Survival

eFigure 1. Study Flow Chart

eFigure 2. Distribution of Patients

eFigure 3. Tumor Markers

eFigure 4. Percentage of Patients Tested by Year of Diagnosis For Each Tumor Marker

eFigure 5. CEA, CA19-9, and CA125 by Sex

eFigure 6. CEA, CA19-9, and CA125 by Histopathology

eFigure 7. Correlation of CEA, CA19-9, and CA125

eFigure 8. Survival Probability for all Patients Stratified by Tumor Markers

eFigure 9. Survival Probability by Grade

eFigure 10. Survival Probability for CEA, CA19-9, and CA125 for Patients Tested Within the First 6 Months From Diagnosis

eFigure 11. Survival Probability for CEA, CA19-9, and CA125 for All Patients vs Metastatic in the Validation Cohort (The Christie NHS Foundation Trust Cohort)

© 2024 Yousef A et al. *JAMA Network Open.*

eFigure 12. Survival Probability After Receiving Chemotherapy Stratified by CEA
eFigure 13. Association of *GNAS* and *KRAS* Somatic Mutations With Tumor Markers

© 2024 Yousef A et al. *JAMA Network Open.*

eAppendix. Supplemental Methods

Survival analyses were performed using the Kaplan-Meier (KM) method with log-rank test. Univariate and multivariable Cox-proportional hazards (CPH) regression analyses were performed to assess associations between clinical factors (serum TM levels, demographics, and patient and disease characteristics) and patient outcomes (OS). KM and CPH analyses modeled TMs individually (model A) as well as in a single aggregate variable indicating the number of elevated TMs (model B). In model B, for TMs, the covariate was an ordinal variable of 0 biomarkers elevated, 1 biomarker elevated, 2 biomarkers elevated, or 3 biomarkers elevated. In both models, sex, race, smoking status, alcohol use, age at diagnosis, tumor histologic findings, tumor grade, and tumor stage were also considered. All variables with $P < .05$ on univariate analysis were included in the multivariable analysis. Differences in tumor histopathological types between patients with elevated vs normal TM levels were assessed using Fisher exact test. Differences in TM levels between tumor grade and gender were assessed using Mann-Whitney U test, while differences in TM levels between tumor histopathology were assessed using Kruskal-Wallis test as TM levels didn't follow Gaussian distribution. Correlation between the different TMs were assessed using Spearman correlation. Frequency of mutations among patients with elevated vs normal TM levels was assessed using Fisher exact test.

A cohort from The Colorectal and Peritoneal Oncology Center, The Christie National Health Service (NHS) Foundation Trust, Manchester, United Kingdom, was used for validation. Between January 2005 and August 2021, patients with histologically confirmed AAs were identified from a prospectively collected database. Patients were discussed in a specialized peritoneal tumor multidisciplinary team (MDT) and all pathology slides reported at external institutions were imported and re-reported by specialist pathologists. Data including demographics, clinicopathological variables, TMs, and survival status were extracted from the database. All patients had pathology reports reviewed; missing data were referenced from the hospital record.

© 2024 Yousef A et al. *JAMA Network Open.*

eTable 1. Validation Cohort Patient Characteristics

Patient Characteristics		All<br>N (100%)
**All**		**216 (100%)**
**Age at diagnosis - median (min, max)**		**59 (21, 80)**
**Sex**		
	**Female**	**126 (58%)**
	**Male**	**90 (42%)**
**Histopathology**		
	**Mucinous**	**141 (65%)**
	**Adenocarcinoma not otherwise specified**	**71 (33%)**
	**Signet ring carcinoma**	**4 (2%)**
**Disease Metastatic State**		
	**Localized Disease (Stage I, II, III)**	**69 (32%)**
	**Metastatic Disease (Stage IV)**	**147 (68%)**
**CEA**		
	**Normal**	**116 (54%)**
	**Elevated**	**67 (31%)**
	**Highly Elevated**	**21 (9%)**
	**Not tested**	**12 (6%)**
**CA19-9**		
	**Normal**	**137 (63%)**
	**Elevated**	**48 (22%)**
	**Highly Elevated**	**19 (9%)**
	**Not tested**	**12 (6%)**
**CA125**		
	**Normal**	**137 (63%)**
	**Elevated**	**46 (21%)**
	**Highly Elevated**	**21 (10%)**
	**Not tested**	**12 (6%)**
**Overall Survival (months)**		
	**Median (min, max)**	**122 [1, 286]**

© 2024 Yousef A et al. *JAMA Network Open.*

eTable 2. Tumor Marker Levels and Cutoffs for the Main Cohort

	CEA	CA 19-9	CA 125
Number of Patients (n)	1331	1132	1165
Cut off Value	> 3.8 ng/mL	> 35 U/mL	> 38 U/mL
Percentage Elevated (%)	46%	24%	17%
Number Elevated	609	268	196
Highly Elevated cut off (top 10th percentile)	> 99.8 ng/mL	> 338.6 U/mL	> 99.0 U/mL
Percentage Highly Elevated (%)	10%	10%	10%
Number Highly Elevated	133	113	116

eTable 3. Number of Test Performed for Each TM per Patient

	CEA	CA 19-9	CA 125
Mode	1	1	1
Approximate Median	4	3	3
Approximate Mean	7	5	5
Min	1	1	1
Max	98	60	36

eTable 4. Tumor Marker Levels and Cutoffs Split by Tumor Grade

	CEA		CA 19-9		CA 125	
Grade	Low Grade	High Grade	Low Grade	High Grade	Low Grade	High Grade
Number of Patients (n)	519	761	486	607	487	636
Cut off Value	> 3.8 ng/mL		> 35 U/mL		> 38 U/mL	
Percentage Elevated (%)	45%	44%	27%	22%	15%	18%
Number Elevated	235	355	129	132	75	113
Highly Elevated cut off (top 10th percentile)	> 99.8 ng/mL		> 338.6 U/mL		> 99.0 U/mL	
Percentage Highly Elevated (%)	10%		10%		10%	
Number Highly Elevated	53	78	41	70	37	76

© 2024 Yousef A et al. *JAMA Network Open.*

eTable 5. CEA Level and Cutoff Split by Histopathology

	CEA				
Grade	Mucinous	Colonic	Goblet	Signet	Goblet & Signet
Number of Patients (n)	693	130	93	221	147
Cut off Value	> 3.8 ng/mL				
Percentage Elevated (%)	56%	59%	30%	69%	50%
Number Elevated	391	77	28	153	73
Mean	64.3	172.8	12.2	44.6	10.5
Std. Deviation	244	593	62	126	28
Median	4.3	5.1	2.7	6.7	3.6

eTable 6. CA19-9 Level and Cutoff Split by Histopathology

	CA19-9				
Grade	Mucinous	Colonic	Goblet	Signet	Goblet & Signet
Number of Patients (n)	630	90	77	180	118
Cut off Value	> 35 U/mL				
Percentage Elevated (%)	36%	39%	10%	43%	21%
Number Elevated	224	35	8	77	25
Mean	272	5829	35	408	72
Std. Deviation	1431	37295	130	1413	390
Median	20	21	13	27	14

© 2024 Yousef A et al. *JAMA Network Open.*

eTable 7. CA125 Level and Cutoff Split by Histopathology

	CA125				
**Grade**	**Mucinous**	**Colonic**	**Goblet**	**Signet**	**Goblet & Signet**
**Number of Patients (n)**	**631**	**84**	**79**	**190**	**130**
**Cut off Value**	**> 38 U/mL**				
**Percentage Elevated (%)**	**25%**	**25%**	**13%**	**38%**	**26%**
**Number Elevated**	**157**	**21**	**10**	**73**	**34**
**Mean**	**34.0**	**59.0**	**21.5**	**59.2**	**44.5**
**Std. Deviation**	**49.6**	**168.4**	**26.7**	**89.8**	**74.5**
**Median**	**15.7**	**15.4**	**12.9**	**21.6**	**17.7**

© 2024 Yousef A et al. *JAMA Network Open.*

eTable 8. Cox proportional hazards regression model A for overall survival (n=1338)

	Univariate Analysis				Multivariate Analysis			
Clinical Characteristics	HR	P value	95 CI%		HR	P value	95 CI%	
Male gender		reference				reference		
Female gender	0.6589	0.0036	0.4977	0.8722	0.78	0.16	0.54	1.1
Race white		reference				Reference		
Race Black or African American	1.9	0.0072	1.19	3.033	2.9	0.0005	1.6	5.1
Race Hispanic/ Latino	1.264	0.3156	0.7999	1.997	2.5	0.0017	1.4	4.5
Race Asian	1.531	0.2717	0.7164	3.272				
Race Others	0.7099	0.6305	0.1758	2.867				
Smoking status Never		reference				reference		
Smoking status Former	1.294	0.1215	0.9338	1.794				
Smoking status Smoker	0.9791	0.9566	0.4575	2.095				
Alcohol use status Never		reference				reference		
Alcohol use status Yes	0.7444	0.0462	0.5569	0.995	1.2	0.45	0.8	1.7
Age at diagnosis	1.015	0.0218	1.002	1.028	1	0.28	0.99	1
Tumor Histopathology Mucinous		reference				reference		
Tumor Histopathology Colonic	2.855	9.32E-06	1.795	4.539	1.9	0.063	0.97	3.7
Tumor Histopathology Goblet	0.2172	0.1297	0.03014	1.565				
Tumor Histopathology Signet	4.278	5.24E-17	3.045	6.009	3.1	1.8E-07	2	4.8
Tumor Histopathology Goblet/Signet	3.228	6.13E-08	2.112	4.933	3.3	4.0E-05	1.9	5.8
Low Grade tumor		reference				reference		
High Grade Tumor	3.782	1.61E-14	2.693	5.31	3.5	1.70E-08	2.3	5.4
Normal CEA		reference				reference		
Elevated CEA	4.997	6.52E-16	3.382	7.382	2.8	0.0001	1.7	4.9
Normal CA19-9		reference				reference		
Elevated CA19-9	3.088	1.16E-11	2.23	4.277	1.5	0.028	1	2.2
Normal CA125		reference				reference		
Elevated CA125	5.936	2.97E-27	4.298	8.198	3.2	1.70E-09	2.2	4.7
Localized Disease		reference				reference		
Metastatic Disease	11.51	1.33E-6	4.275	30.99	9.8	0.0017	2.4	41

© 2024 Yousef A et al. *JAMA Network Open.*

eTable 9. Cox proportional hazards regression model B for overall survival (n=1338)

	Univariate Analysis				Multivariate Analysis			
Clinical Characteristics	HR	P value	95 CI%		HR	P value	95 CI%	
Male gender		reference				reference		
Female gender	0.6589	0.0036	0.4977	0.8722	0.69	0.017	0.51	0.94
Race white		reference				reference		
Race Black or African American	1.9	0.0072	1.19	3.033	1.9	0.013	1.1	3.2
Race Hispanic/ Latino	1.264	0.3156	0.7999	1.997	1.9	0.013	1.1	3.1
Race Asian	1.531	0.2717	0.7164	3.272	1.2	0.64	0.5	3.1
Race Others	0.7099	0.6305	0.1758	2.867	1	0.98	0.25	4.2
Smoking status Never		reference				reference		
Smoking status Former	1.294	0.1215	0.9338	1.794				
Smoking status Smoker	0.9791	0.9566	0.4575	2.095				
Alcohol use status Never		reference				reference		
Alcohol use status Yes	0.7444	0.0462	0.5569	0.995	1	0.99	0.73	1.4
Age at diagnosis	1.016	0.0127	1.003	1.029	1	0.5	0.99	1
Tumor Histopathology Mucinous		reference				reference		
Tumor Histopathology Colonic	2.855	9.32E-06	1.795	4.539	2	0.0071	1.2	3.4
Tumor Histopathology Goblet	0.2172	0.1297	0.03014	1.565				
Tumor Histopathology Signet	4.278	5.24E-17	3.045	6.009	3	7.60E-09	2.1	4.4
Tumor Histopathology Goblet/Signet	3.228	6.13E-08	2.112	4.933	3.1	6.10E-06	1.9	5
Low Grade tumor		reference				reference		
High Grade Tumor	3.782	1.61E-14	2.693	5.31	3.4	1.10E-09	2.3	5
Normal level of all the 3 TMs		reference				reference		
Elevated one TM (Ref: 0 elevated)	6.831	5.13E-11	3.85	12.12	4	3.6E-05	2.1	7.7
Elevated two TM (Ref: 1 elevated)	1.484	0.02	1.1	2.1	1.6	0.012	1.1	2.3
Elevated three TM (Ref: 2 elevated)	1.575	0.02	1.1	2.3	1.7	0.011	1.1	2.5
Elevated three TM (Ref: 0 elevated)	15.97	3.30E-19	8.709	29.27	11	1.0E-11	5.4	21
Localized Disease		reference				reference		
Metastatic Disease	11.51	1.33E-06	4.275	30.99	9.3	0.0002	2.9	30

© 2024 Yousef A et al. *JAMA Network Open.*

Figure Legends

eFigure 1. Flowchart diagram showing cohort patients selection. Abbreviations include MDACC (MD Anderson Cancer Center), HER (Electronic Health Records).

eFigure 2. (A) Distribution of all patients in our cohort by year of diagnosis. (B) Distribution of all patients in our cohort by tumor histopathological grade. (C) Distribution of all patients in our cohort by tumor histopathological grade binary.

eFigure 3. (A) Bar plot showing number of patients with normal, elevated, and highly elevated levels of CEA, CA19-9, and CA125. (B) Proportionate Venn diagram showing the overlapping of elevated (highly elevated included) levels of the three tumor markers for all patients tested for the 3 TMs. (C) Proportionate Venn diagram showing the overlapping of elevated (highly elevated included) levels of the three tumor markers for patients with metastatic disease tested for the 3 TMs.

eFigure 4. Spider plot showing % of patients tested for each tumor marker at the time of diagnosis over the last 8 years.

eFigure 5. Violin plot showing the distribution of all patients CEA, CA19-9, and CA125 tumor markers levels split by patients sex, lines represent median levels

eFigure 6. (A) Violin plot showing the distribution of all patients CEA tumor markers levels split by tumor histopathology, lines represent median levels. (B) Violin plot showing the distribution of all patients CA19-9 tumor markers levels split by tumor histopathology, lines represent median levels. (C) Violin plot showing the distribution of all patients CA125 tumor markers levels split by tumor histopathology, lines represent median levels.

eFigure 7. (A) Scattered plot for patients who had CEA (on X-axis) and CA19-9 (on Y-axis) measured on the same day showing the correlation between both tumor markers. (B) Scattered plot for patients who had CEA (on X-axis) and CA125 (on Y-axis) measured on the same day showing the correlation between both tumor markers (C) Scattered plot for patients who had CA19-9 (on X-axis) and CA125 (on Y-axis) measured on the same day showing the correlation between both tumor markers. Each point represents one measurement on one day for one patient. Non parametric Spearman's correlation was used to measure the degree of association, (all $p<0.0001$)

eFigure 8. (A) KM survival plot of all patients with normal, elevated, and highly elevated levels of CEA. (B) KM survival plot of all patients with normal, elevated, and highly elevated levels of CA19-9. (C) KM survival plot of all patients with normal, elevated, and highly elevated levels of CA125.

eFigure 9. (A) KM survival plot of patients with low grade tumor for normal, elevated, and highly elevated levels of CA19-9. (B) KM survival plot of patients with high grade tumor for normal, elevated, and highly elevated levels of CA19-9. (C) KM survival plot of patients with low grade tumor for normal, elevated, and highly elevated levels of CA125. (D) KM survival plot of patients with high grade tumor for normal, elevated, and highly elevated levels of CA125.

eFigure 10. (A) KM survival plot of all patients with normal, elevated, and highly elevated levels of CEA measured within the initial six months from the date of diagnosis. (B) KM

© 2024 Yousef A et al. *JAMA Network Open.*

survival plot of all patients with normal, elevated, and highly elevated levels of CA19-9 measured within the initial six months from the date of diagnosis. (C) KM survival plot of all patients with normal, elevated, and highly elevated levels of CA125 measured within the initial six months from the date of diagnosis. (D) KM survival plot of all patients with number of elevated tumor markers measured within the initial six months from the date of diagnosis. (E) Forest plot for multivariable analysis showing HR for death in the subset of patients who had their tumor markers measured within the initial six months from the date of diagnosis.

eFigure 11. The validation cohort (The Christie NHS Foundation Trust Cohort) (A) KM survival plot of all patients with normal, elevated, and highly elevated levels of CEA. (B) KM survival plot of metastatic disease patients with normal, elevated, and highly elevated levels of CEA (C) KM survival plot of all patients with normal, elevated, and highly elevated levels of CA19-9. (D) KM survival plot of metastatic disease patients with normal, elevated, and highly elevated levels of CA19-9. (E) KM survival plot of all patients with normal, elevated, and highly elevated levels of CA125. (F) KM survival plot of metastatic disease patients with normal, elevated, and highly elevated levels of CA125.

eFigure 12. KM survival plot for the subset of patients (n=121) who received chemotherapy at MDACC showing the difference in OS between patients who had ≥ 2ng/mL elevation in CEA and patients who had CEA levels normalized or remained stable.

eFigure 13. (A-C) Bar graphs showing the percentage of patients with elevated CEA (red), CA19-9 (blue), and CA125 (green) in mutant and wildtype *KRAS*, *GNAS*, and *TP53*. (D) Scattered plots for *KRAS* mutant vs wildtype with CA19-9 and CA125 levels, lines represents the median levels. (E) Scattered plots for *GNAS* mutant vs wildtype with CA19-9 and CA125 levels, lines represents the median levels.

© 2024 Yousef A et al. *JAMA Network Open.*

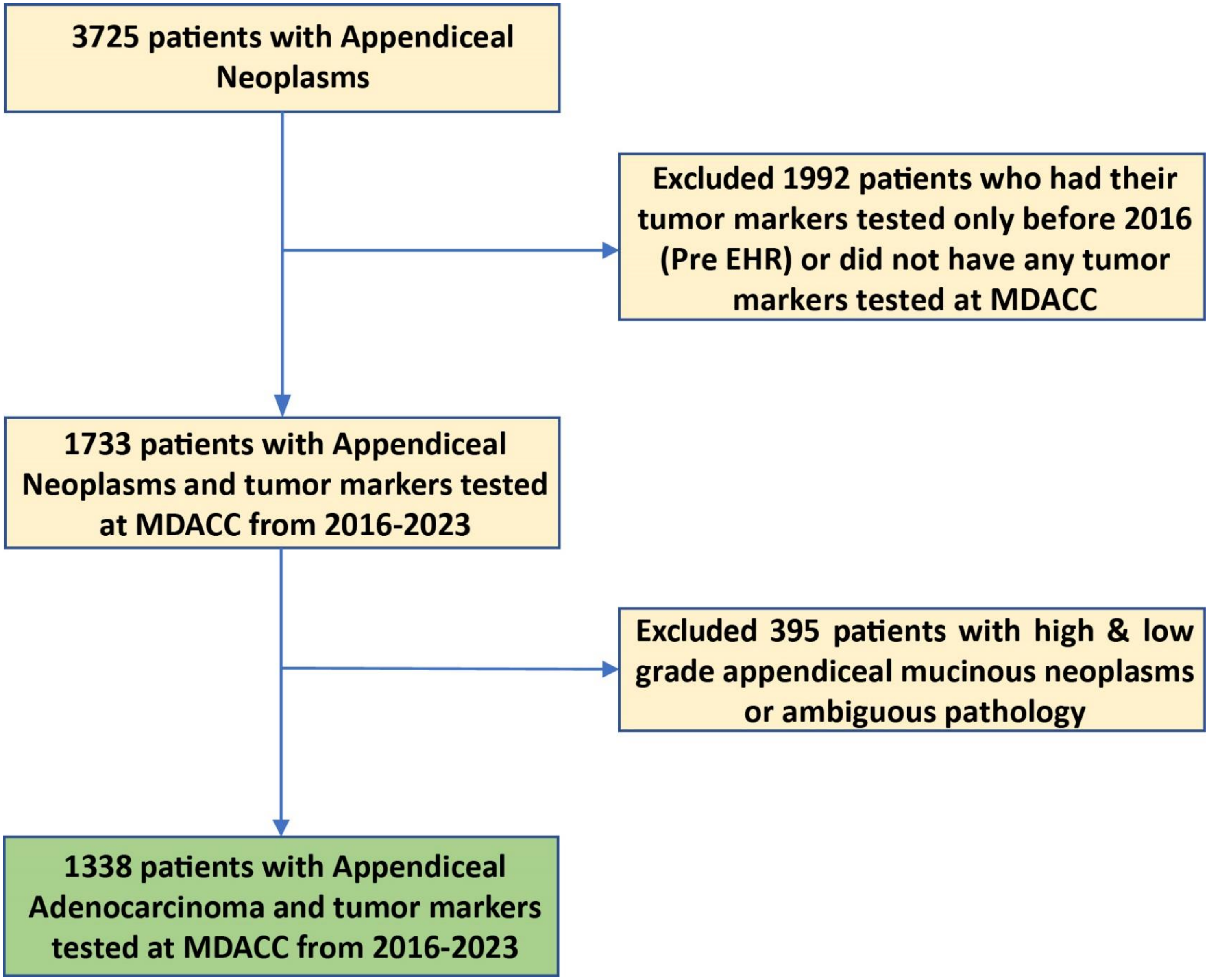

eFigure 1. Study Flow Chart

© 2024 Yousef A et al. *JAMA Network Open.*

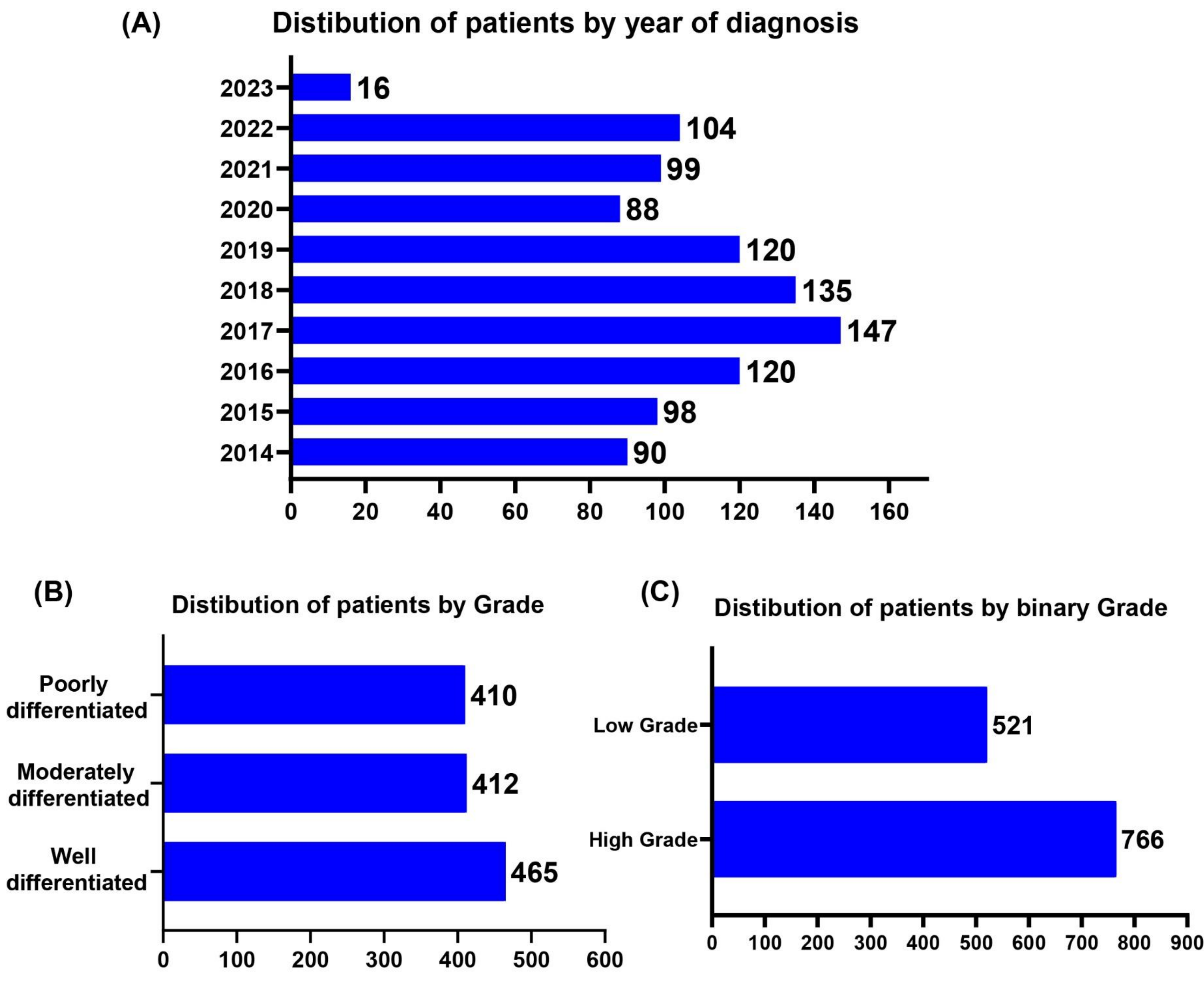

eFigure 2. Distribution of Patients

© 2024 Yousef A et al. *JAMA Network Open.*

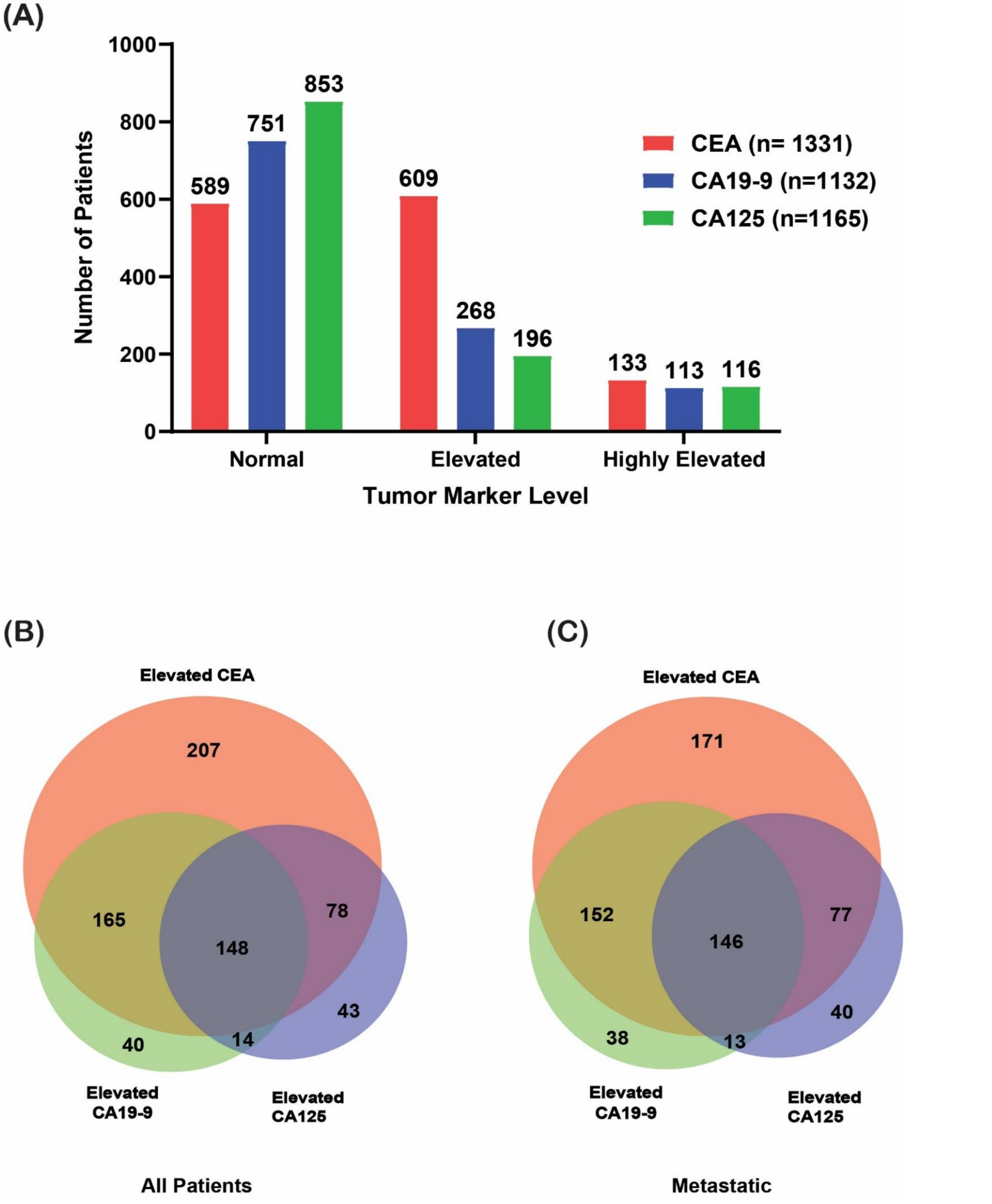

eFigure 3. Tumor Markers

© 2024 Yousef A et al. *JAMA Network Open.*

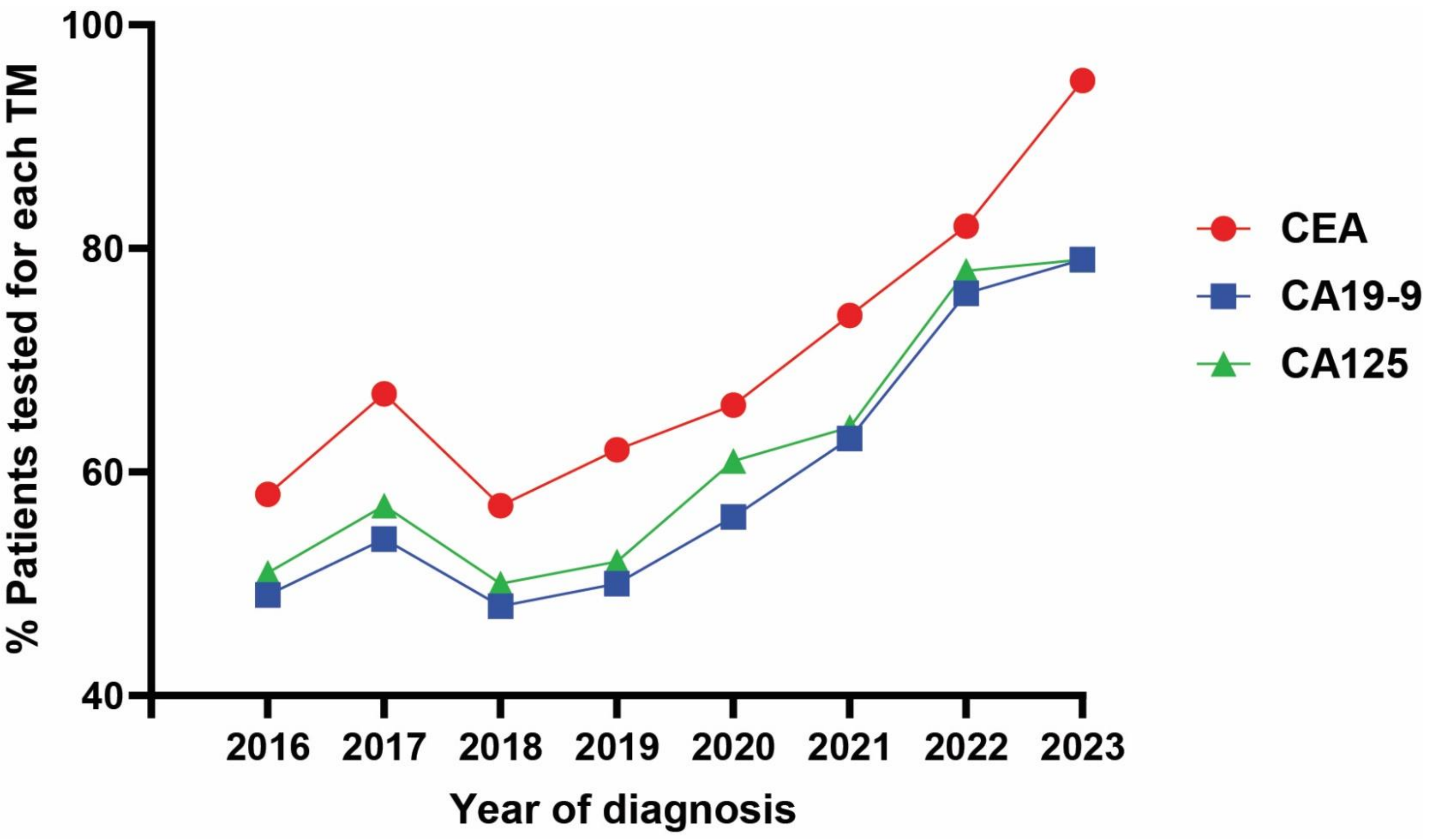

eFigure 4. Percentage of Patients Tested by Year of Diagnosis For Each Tumor Marker

© 2024 Yousef A et al. *JAMA Network Open.*

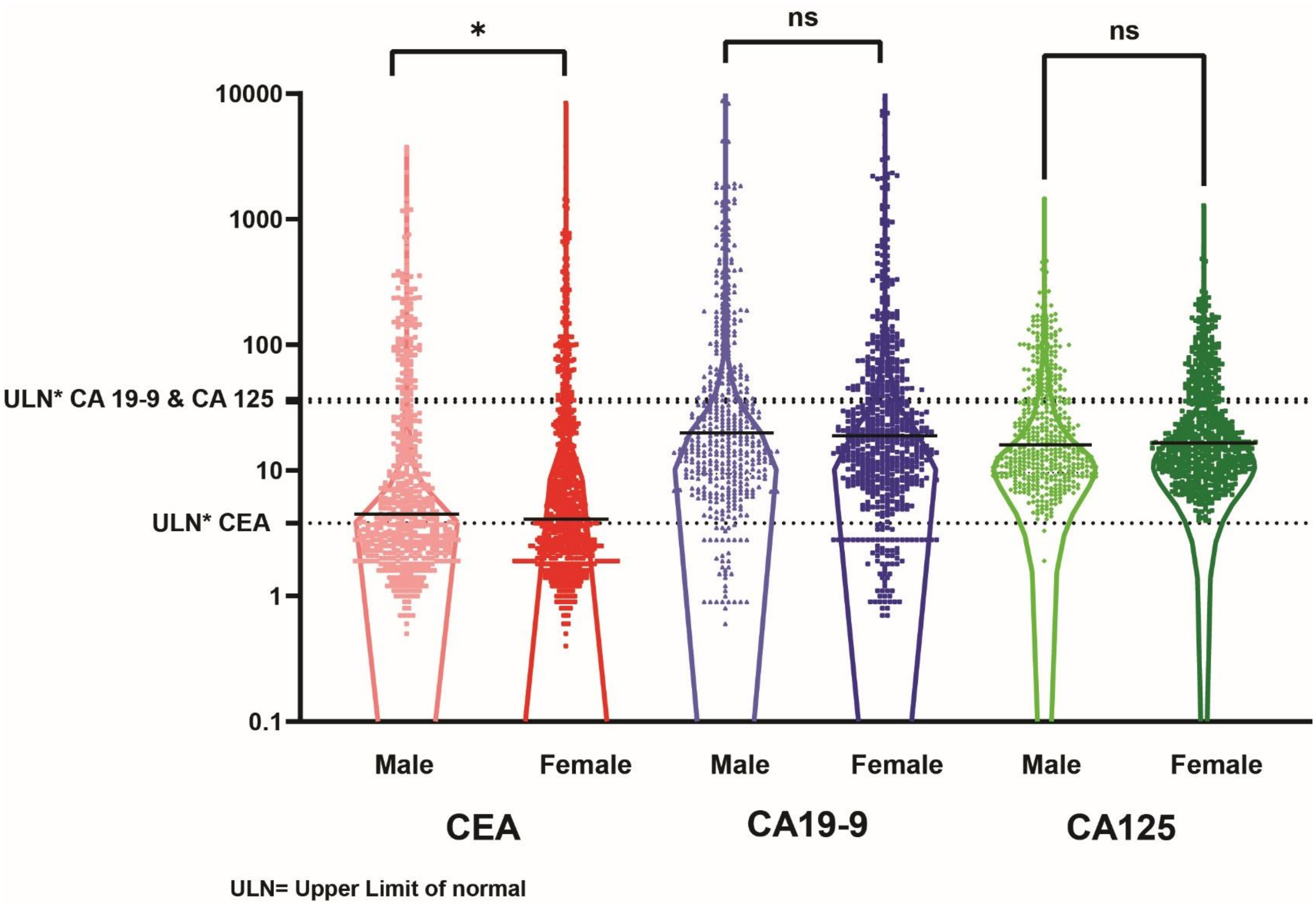

eFigure 5. CEA, CA19-9, and CA125 by Sex

© 2024 Yousef A et al. *JAMA Network Open.*

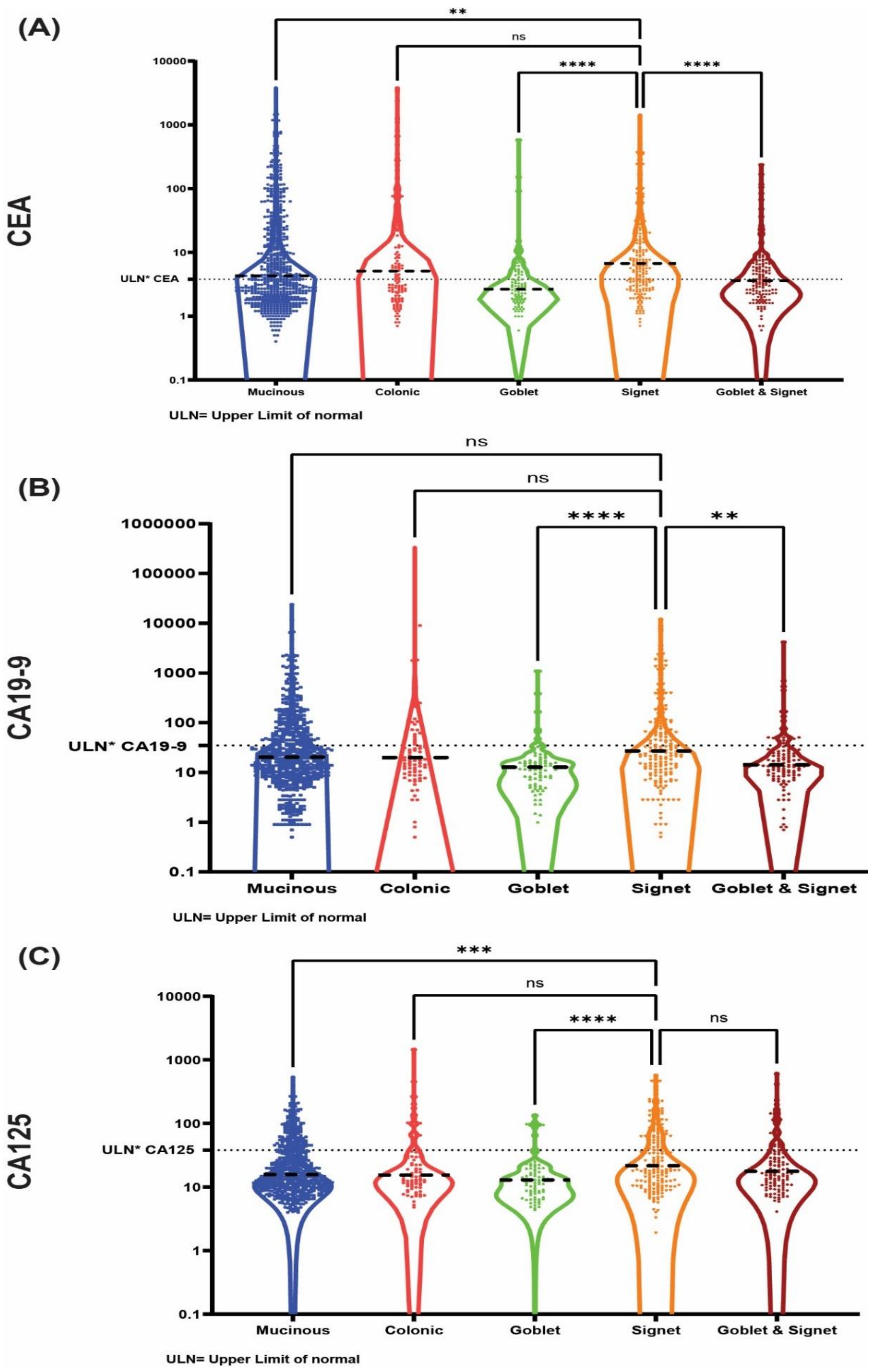

eFigure 6. CEA, CA19-9, and CA125 by histopathology

© 2024 Yousef A et al. *JAMA Network Open.*

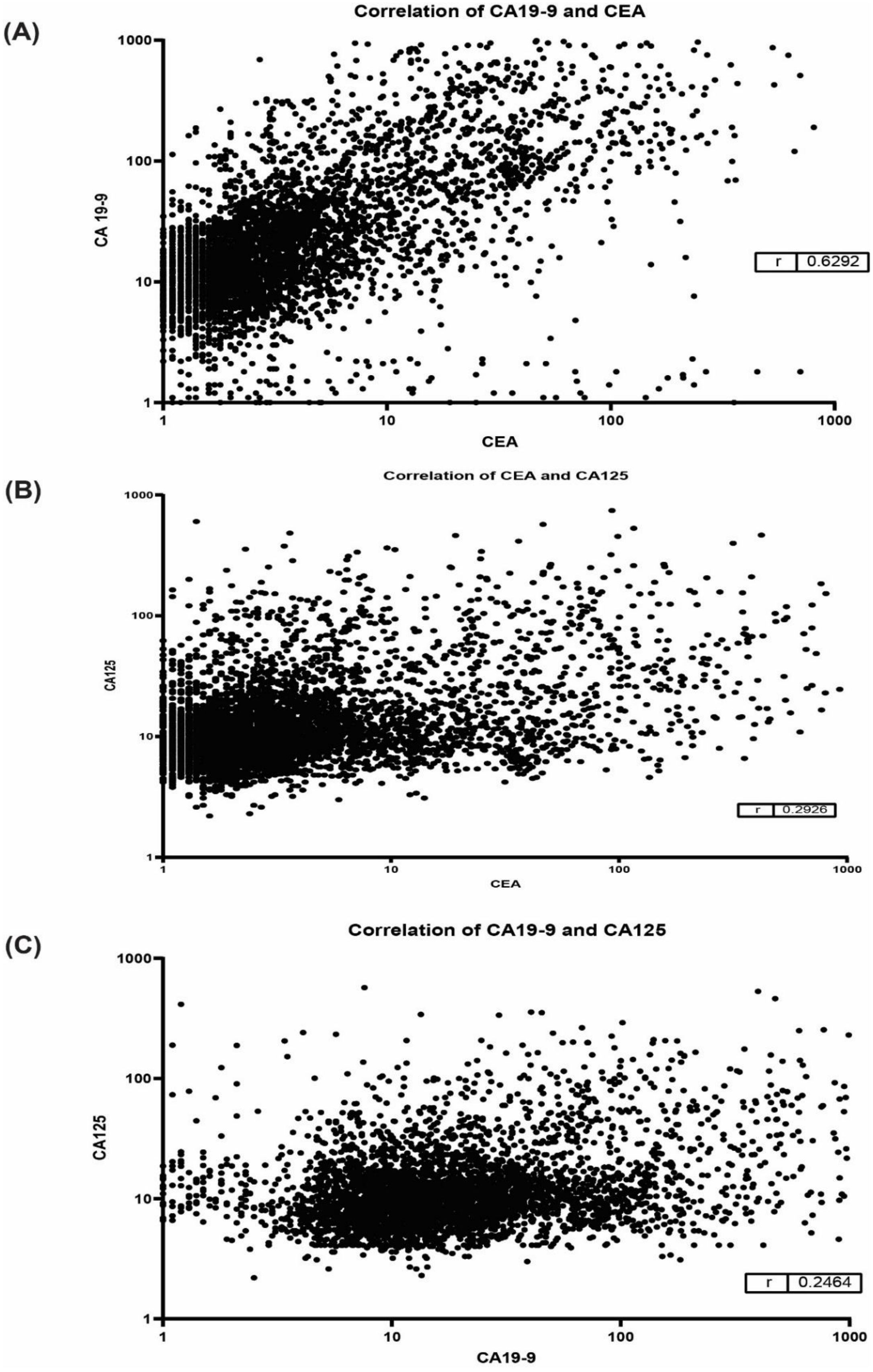

eFigure 7. Correlation of CEA, CA19-9, and CA125

© 2024 Yousef A et al. *JAMA Network Open.*

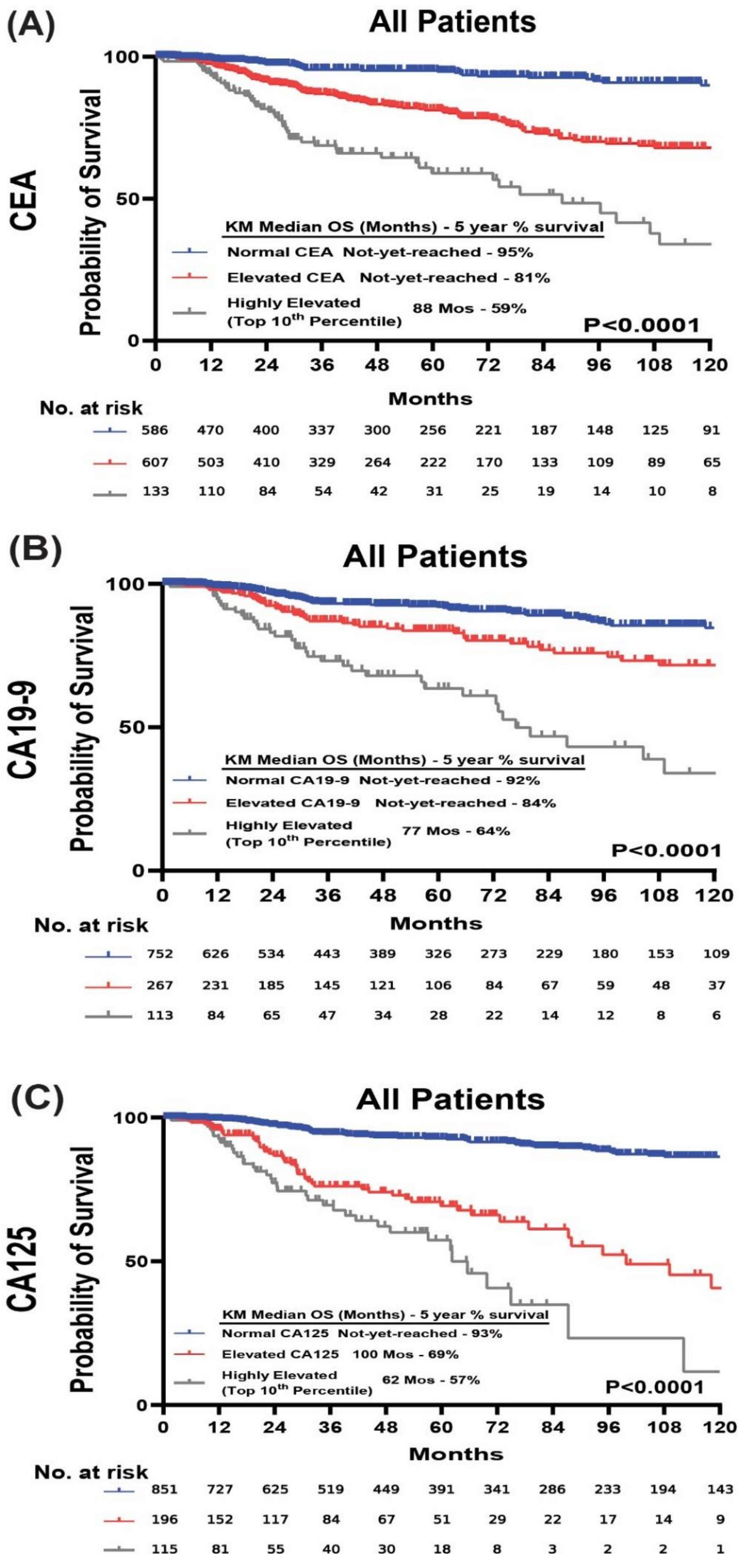

eFigure 8. Survival Probability for all patients stratified by tumor markers

© 2024 Yousef A et al. *JAMA Network Open.*

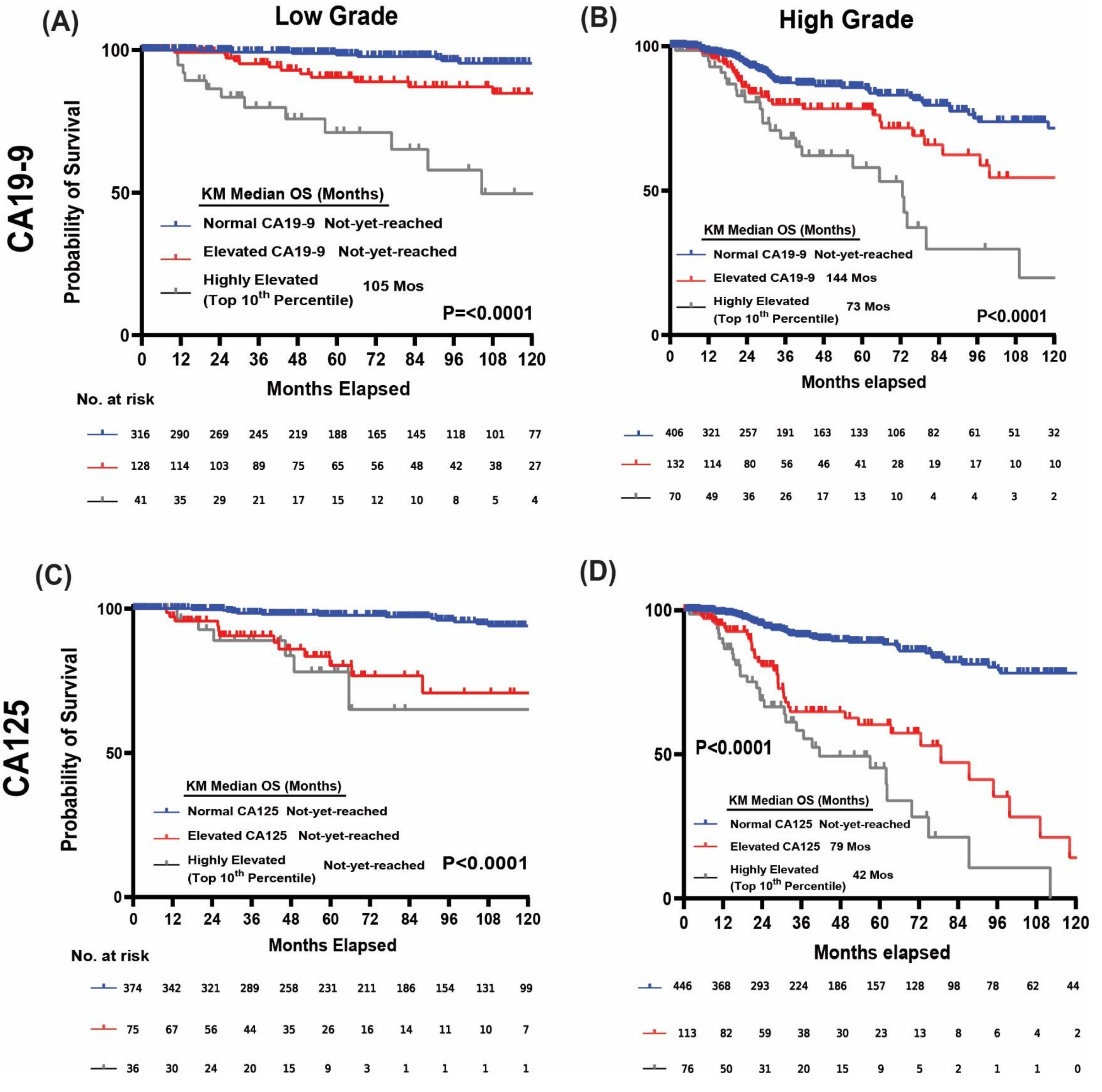

eFigure 9. Survival Probability by Grade

© 2024 Yousef A et al. *JAMA Network Open.*

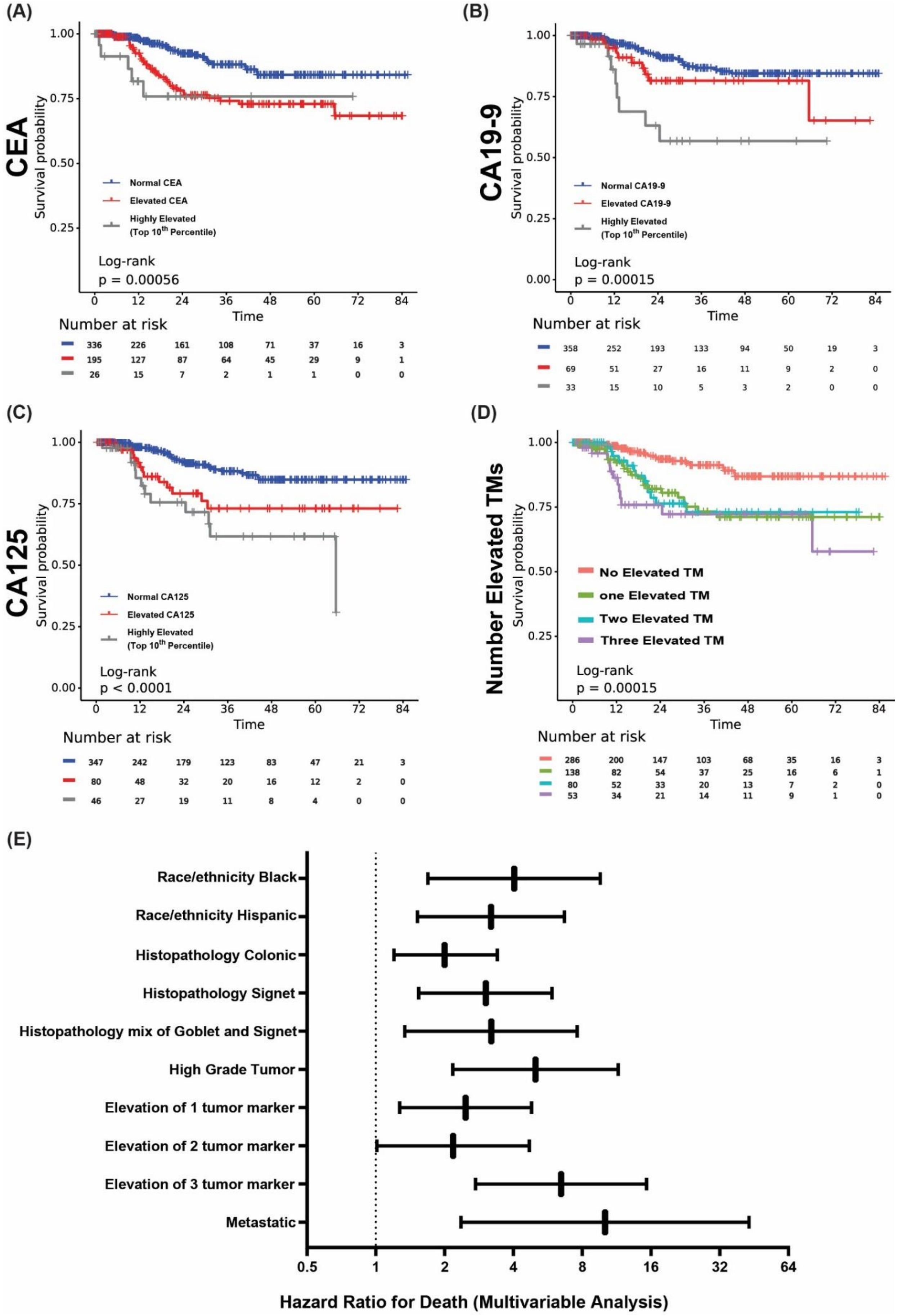

eFigure 10. Survival Probability for CEA, CA19-9, and CA125 for patients tested within the First 6 months from diagnosis

© 2024 Yousef A et al. *JAMA Network Open.*

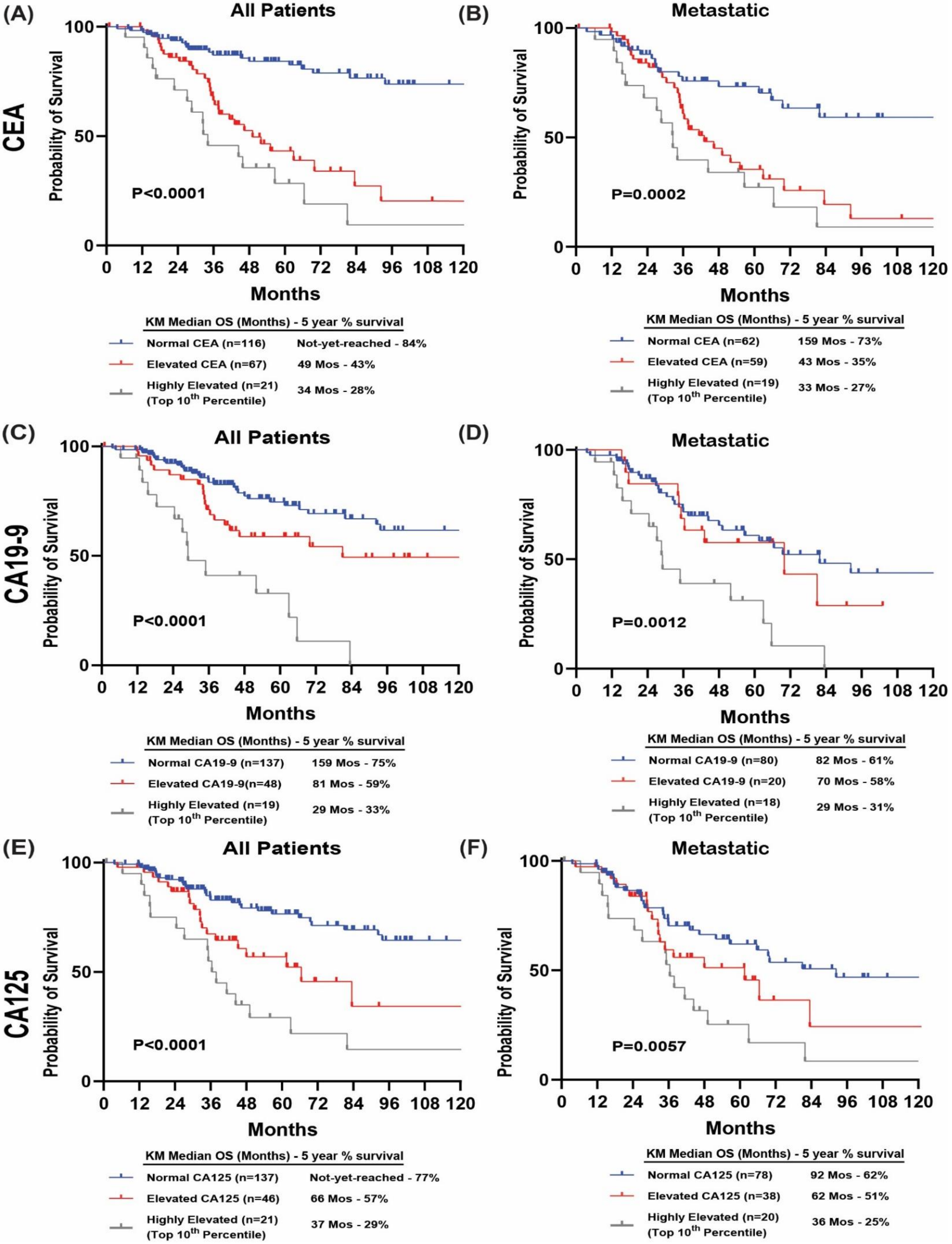

eFigure 11. Survival Probability for CEA, CA19-9, and CA125 for All Patients vs Metastatic in the validation cohort

© 2024 Yousef A et al. *JAMA Network Open.*

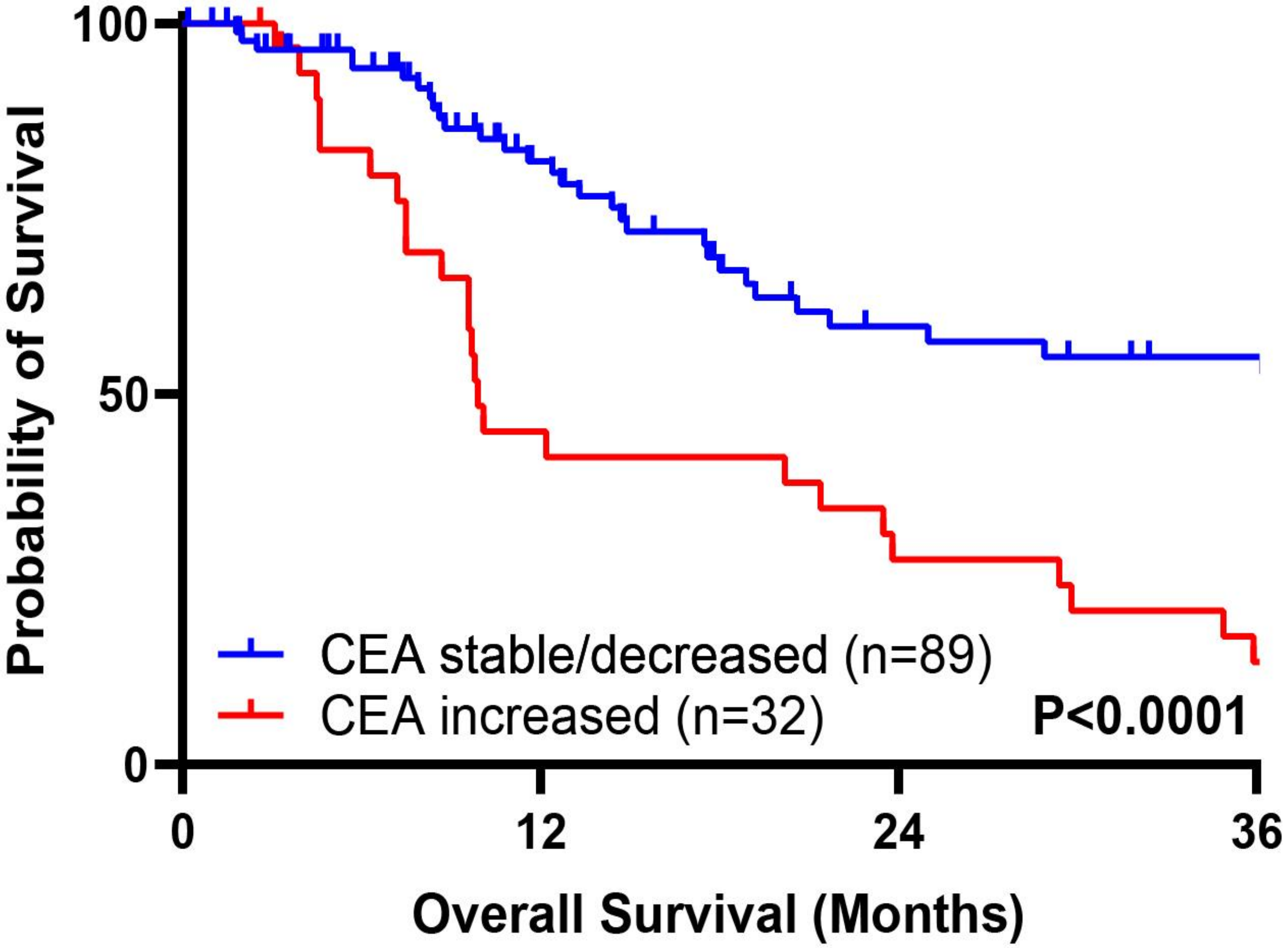

eFigure 12. Survival probability after receiving chemotherapy stratified by CEA

© 2024 Yousef A et al. *JAMA Network Open.*

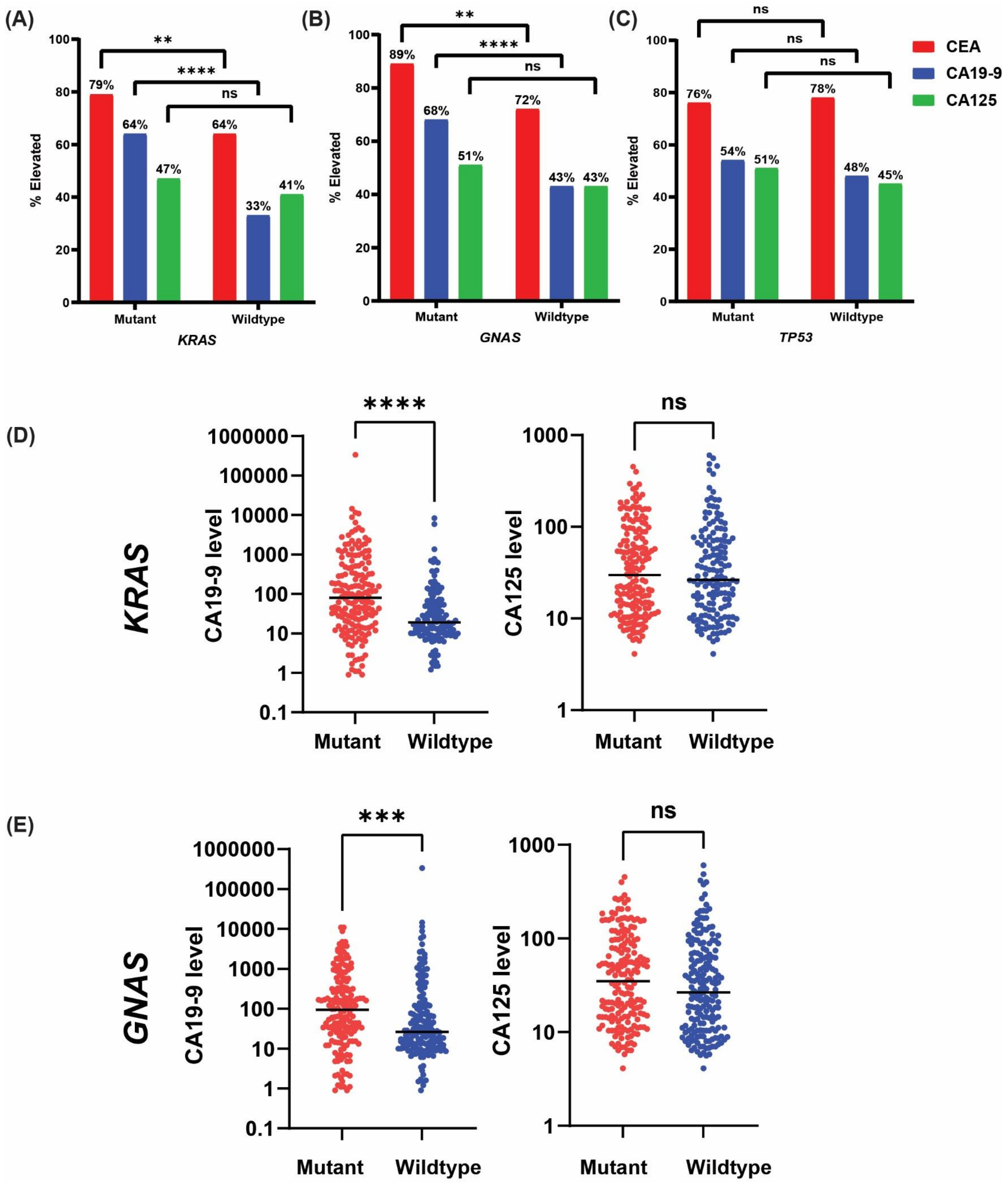

eFigure 13. Association of *GNAS* and *KRAS* Somatic Mutations With Tumor Markers
