# Supplementary material for: Serum Tumor Markers and Outcomes in Patients With Appendiceal Adenocarcinoma
Source: JAMA Netw Open. 2024 Feb 28;7(2):e240260. doi: 10.1001/jamanetworkopen.2024.0260 (PMC10902735; doi:10.1001/jamanetworkopen.2024.0260)
Supplement: Supplement 2. — Data Sharing Statement [file jamanetwopen-e240260-s002.pdf]

Data Sharing Statement

Yousef. Serum Tumor Markers and Outcomes in Patients With Appendiceal Adenocarcinoma. *JAMA Netw Open*. Published February 23, 2024. doi:10.1001/jamanetworkopen.2024.0260

Data
Data available: Yes
Data types: Deidentified participant data
How to access data: The data generated in this study are included in the supplementary tables or otherwise available upon request to the corresponding author
When available: With publication

Supporting Documents
Document types: None

Additional Information
Who can access the data: The data generated in this study are included in the supplementary tables or otherwise available upon request to the corresponding author
Types of analyses: any purpose
Mechanisms of data availability: after approval of a proposal
